# A Review on Understanding and Strengthening of Active Species in Titanium Silicalite‐1 (TS‐1) Catalysts

**DOI:** 10.1002/advs.202512531

**Published:** 2025-11-07

**Authors:** Yan Xue, Shuling Liu, Jiaqi Wang, Huanhuan Zhang, Huijuan Wei, Yanyan Liu, Xiangyu Wang, Jinjun Tian, Jianchun Jiang, Baojun Li

**Affiliations:** ^1^ Nanyang Institute of Technology 80 Changjiang Road Nanyang 473000 P. R. China; ^2^ College of Chemistry Zhengzhou University 100 Science Road Zhengzhou 450001 P. R. China; ^3^ College of Science Henan Agricultural University 95 Wenhua Road Zhengzhou 450002 P. R. China; ^4^ Institute of Chemical Industry of Forest Products, CAF National Engineering Lab for Biomass Chemical Utilization 16 Suojinwucun Nanjing 210042 P. R. China

**Keywords:** framework Ti, reaction pathways, tetra‐coordinated TiO_4_, Ti active species, titanosilicate‐1 (TS‐1)

## Abstract

Titanium silicalite‐1 (TS‐1) zeolite stands out as a top‐tier catalyst for producing key chemical intermediates under mild conditions. This paper briefly summarizes its applications in oxidation reactions such as ammoximation and epoxidation, and explores the various reaction pathways dominated by Ti active species. Its catalytic oxidation performance depend upon the content and coordination of Ti active species. Strategies for designing highly efficient TS‐1 catalysts focus on regulating its structure and active species by adjusting synthesis conditions. Balancing hydrolysis rates of titanium and silicon sources facilitates more framework Ti species and little anatase TiO_2_. Nucleation and crystallization can be controlled to adjust the microenvironment around the active sites by such as pH control, using modifiers, and tuning crystall pathways. Effective regulation of the crystallization process is conducive to the generation of new coordination Ti species beyond the tetra‐coordinated TiO_4_. By employing bases and templates, Ti species in TS‐1 can be redistributed via dissolution‐recrystallization process. Additionally, the introduction of heteroatoms can alter the electronic state of Ti species. These strategies can convert extra‐framework titanium into framework titanium or highly coordinated titanium species, thereby introduce mesopores in TS‐1 crystals. The design of TS‐1 zeolites with diverse active species demonstrates promising industrial applications.

## Graphical abstract

The hydrolysis rate matching strategy, crystallization rate matching strategy of the titanium and silicon sources, dissolution‐recrystallization strategy in the synthesis process can be designed to regulate the microenvironment of active centers to design TS‐1 enriched in Ti active species.

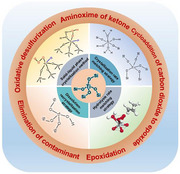

## 1 Introduction

Cyclohexanone oxime, propylene oxide (PO), caprolactam and other important chemical intermediates as organic chemical raw materials are widely used in textile, automobile, electronics, machinery and other fields.^[^ ^1^, ^2^, ^3^ ^]^ The cyclohexanone hydroxylamine process was used for the early industrial synthesis of cyclohexanone oxime. However, this strategy produces significant amounts of low‐value ammonium sulfate.^[^ ^4^, ^5^ ^]^ The hydroperoxidation and chlorohydrin processes were traditionally used to produce propylene oxide. However, Cl_2_ and Ca(OH)_2_ or NaOH are always required for the chlorohydrin process.^[^ ^6^ ^]^ Therefore, significant quantities of chlorinated organic waste liquid are generated by the production of propylene oxide, causing serious environmental problems.

**Figure 1:**
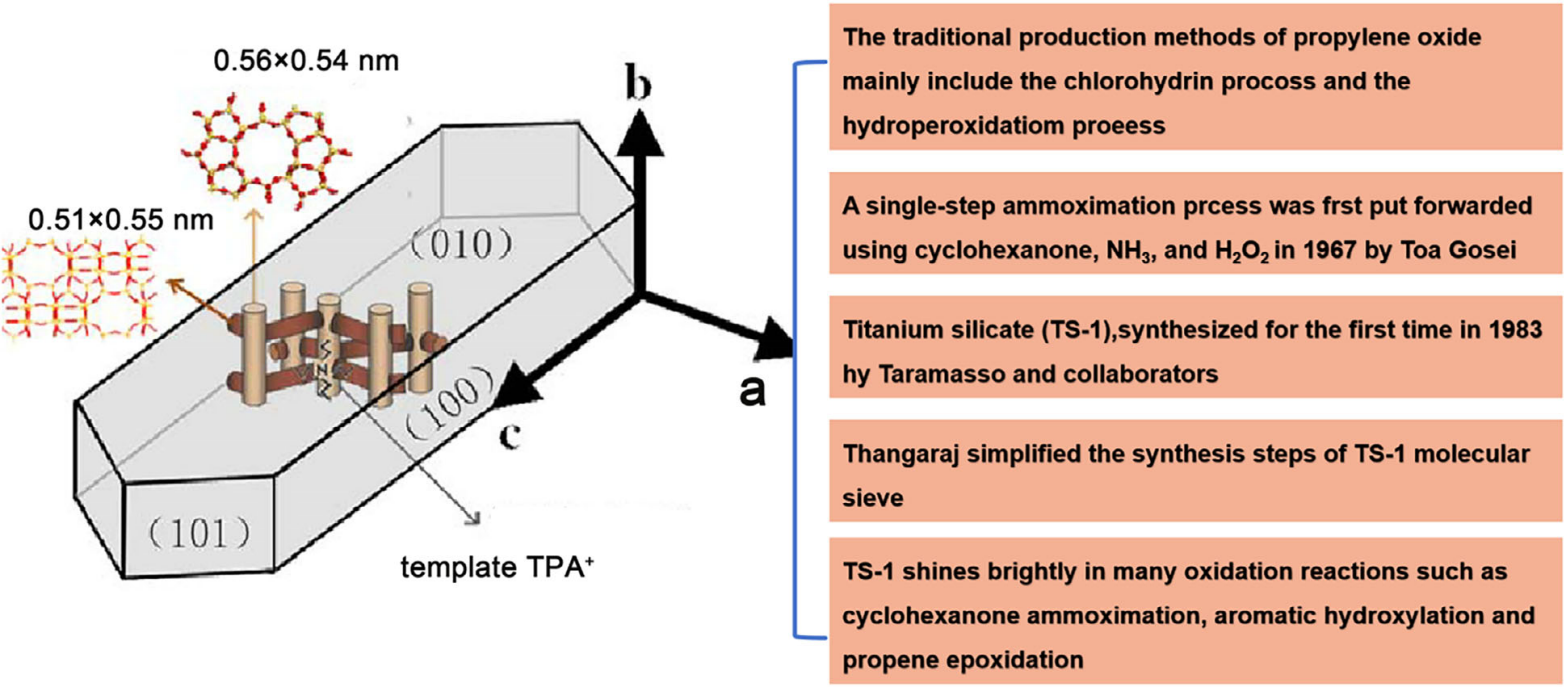
Pore structure and development history of TS‐1.

The production methods of these chemical intermediates inevitably have some shortcomings, such as long production processes, high costs, serious equipment corrosion, the formation of many low‐value by‐products, and environmental pollution. This means the production capacities of these intermediates were far below the social demand. Significant effort has been made to develop efficient and green production processes for chemical intermediates such as cyclohexanone oxime, caprolactam, and propylene oxide. Toa Gosei Chemical Industry first proposed the concept of performing ammoximation in a single step utilizing NH_3_, cyclohexanone, and H_2_O_2_ in 1967.^[^ ^7^ ^]^ Titanosilicate‐1 (TS‐1) was first synthesized by Taramasso and coworkers in 1983 and was used as a catalyst to successfully achieve this process.^[^ ^8^ ^]^ TS‐1 has been rapidly employed as a satisfactory catalyst in aromatic hydroxylation, ketone ammoximation, propylene epoxidation, and other oxidation reactions.^[^ ^9^, ^10^, ^11^ ^]^ Subsequently, Thangaraj simplified the steps required for TS‐1 molecular sieve synthesis by employing a relatively slow hydrolysis rate and utilizing tetra butyl orthotitanate (TBOT) to supply the Ti source^[^ ^12^ ^]^ After nearly 40 years of research, the processes used to prepare TS‐1 molecular sieves have become increasingly advanced, and the activity of TS‐1 in selective catalysis has been increasingly improved.^[^ ^13^ ^]^ TS‐1 is synthesized by introducing transition metal titanium into the framework of silicalite‐1. This material belongs to heteroatom zeolite molecular sieves and has MFI topology. TS‐1 has a 3D pore structure, a linear ten‐membered ring parallel to the b‐axis, and an S‐shaped ten‐membered ring channel parallel to the a‐axis, as shown in **Figure** **1**. The ring oriented parallel to the b‐axis exhibits an aperture of 0.54 nm × 0.56 nm, whereas the ring channel parallel to the a‐axis features an aperture of 0.51 nm × 0.55 nm. In TS‐1 molecular sieves, Ti atoms enter the molecular sieve framework and partly replaces some of the Si atoms on the silicalite‐1 (S‐1) framework. The porous structure and TiO_x_ species within TS‐1 zeolite endow it with excellent catalytic oxidation properties, superb shape selectivity, ideal hydrophobicity, high total surface area, and ion exchange capability. The catalytic oxidation properties of TS‐1 are mainly determined by the TiO_x_ species situated on the zeolite lattice.

**Figure 2:**
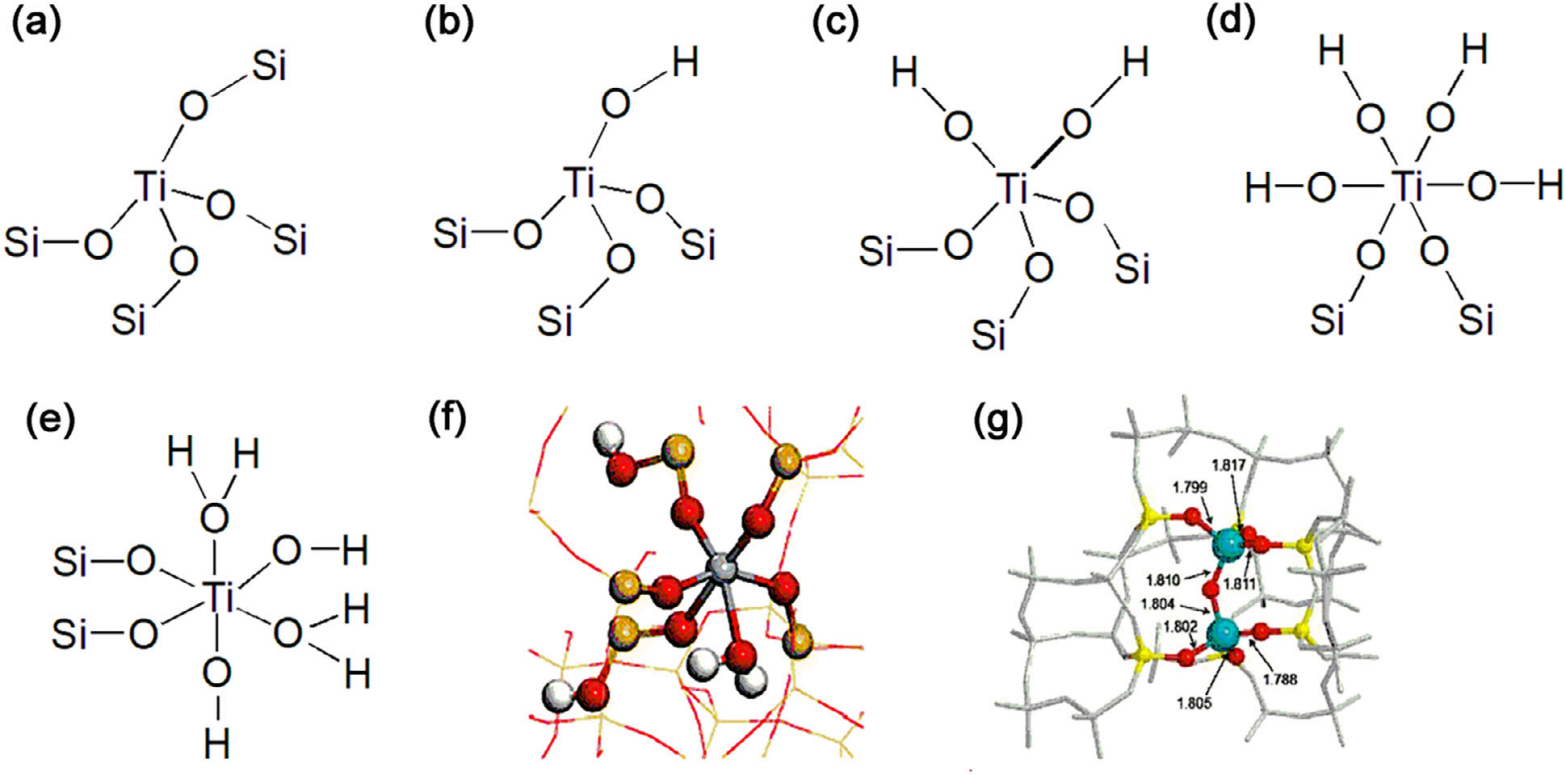
Proposed titanium speices: a) close site, b) open site of tetra‐coordinated Ti (TiO_4_), c) penta‐coordinated Ti (TiO_5_), d,e) hexa‐coordinated Ti (TiO_6_) in TS‐1, f) Proposed structure of the Ti(OH_2_)(OSi)_3_(OSiOH)_2_ by DFT. Reproduced with permission.^[^ ^23^ ^]^ Copyright 2025, Royal Society of Chemistry, g) Resting state and active species proposed in TS‐1 by DFT calculations (blue spheres, Ti; red spheres, O; white spheres, H). Reproduced with permission.^[^ ^25^ ^]^ Copyright 2020, John Wiley & Sons, Inc.

TS‐1 molecular sieves containing various TiO_x_ species, including tetra‐coordinated Ti [TiO_4_], penta‐coordinated Ti [TiO_5_], hexa‐coordinated Ti [TiO_6_] or extra‐framework Ti have been identified and reported.^[^ ^14^, ^15^ ^]^ The tetra‐coordinated Ti [TiO_4_] as the framework Ti species in the lattice of TS‐1 molecular sieve has a tetravalent or forms a tetrahedral coordination structure with four oxygen atoms (TiO_4_). Most tetrahedral coordinated Ti has a perfect “closed” or defect “open” structure. The perfectly “closed” tetrapodal Ti (OSi)_4_ contains four Ti─O─Si bonds (**Figure** **2**a), also known as the framework Ti. The defect “open” TiO_4_ have been reported include tripodal Ti (OSi)_3_(OH) with three Ti─O─Si bonds and one Ti─O─H bond (Figure 2b).^[^ ^16^, ^17^, ^18^ ^]^ For selective oxidation, the perfect “closed” site is generally viewed as the TS‐1 catalytic active species. The penta‐coordinated Ti (TiO_5_) species, as well as a defective “open” Ti(OSi)_3_(OH)_2_ (Figure 2c) structure containing three Ti─O─Si bonds along with two Ti─O─H bonds.^[^ ^16^, ^17^ ^]^ The hexa‐coordinated Ti species (TiO_6_) as another major Ti active species showing octahedrally coordination structures have been reported contain Ti(OH)_4_(OSi)_2_ (Figure 2d),^[^ ^19^ ^]^ and Ti(OH)_2_(H_2_O)_2_(OSi)_2_^[^ ^20^, ^21^, ^22^ ^]^ (Figure 2e). Recently, a novel hexa‐coordinated Ti species, Ti(OH_2_)(OSi)_3_(OSiOH)_2_, is uncovered by Yu and coworks.^[^ ^23^ ^]^ This unique hexa‐coordinated Ti species (Figure 2f) features two Ti─O─Si─OH bonds, differing from the conventional TiO_6_ Ti(H_2_O)_2_(OH)_2_(OSi)_2_. In most cases, it is inevitable that the presence of extra framework Ti species such as rutile and amorphous TiO_2_. Because they can accelerate the breakdown of H_2_O_2_, these species are very detrimental to catalytic oxidation reactions.

Ti species with different coordination forms and structures exhibit different catalytic performance in oxidation reactions. For selective oxidation reactions, tetra‐coordinated Ti is the primary active species of TS‐1 catalysts. However, the presence of some penta‐ and hexa‐ coordinated Ti can lead to enhanced catalytic performance.^[^ ^16^, ^23^ ^]^ These different coordinated Ti species are highly active for the epoxidation of propylene.^[^ ^24^, ^25^, ^26^ ^]^ Penta‐coordinated Ti have been confirmed to be more active than tetra‐coordinated Ti. In particular, Ti(OH)_4_(OSi)_2_ shows a greater level of activity than Ti(OH)_2_(OSi)_3_. Additionally, the catalytic performance of Ti(OH)_4_(OSi)_2_ is roughly 8.7 times higher than that of the commonly recognized tetra‐coordinated framework Ti(OSi)_4_ sites.^[^ ^24^ ^]^

Signorile et al.^[^ ^25^ ^]^ proposed a model involving Ti defective species with higher nuclearity, mainly concerning at least two Ti atoms and has a dimeric tetra‐coordinated Ti structure. The binuclear Ti species in TS‐1 (Figure 2g)^[^ ^25^ ^]^ have been strongly confirmed by ^17^O NMR spectroscopic studies and DFT calculations, containing two Ti atoms and can activate H_2_O_2_. Therefore, binuclear Ti species are regarded as highly effcient active species for propylene epoxidation.

In TS‐1 zeolite, the catalytic activity is intricately linked to the type, coordination state, and defects of titanium (Ti) species. Currently, our grasp of these Ti species remains somewhat limited, with certain unknown variants yet to be identified. By integrating advanced characterization techniques like UV Raman spectroscopy and X‐ray absorption spectroscopy (XAS) with theoretical computations such as density functional theory (DFT), to discover and comprehend a wider array of novel Ti species, along with their detailed structural characteristics.

TS‐1 holds significant importance in the field of fine chemical production, yielding enormous economic benefits. The industrial utilization of TS‐1 molecular sieve faces many difficulties and challenges now. One, only a small amount of titanium can become active Ti species during the synthesis process. Second, TS‐1 molecular sieves have small pore sizes, and many oxidation reactions can only occur on their surface. Enhancing the quantity of active titanium species and enlarging the pore size of the TS‐1 molecular sieve would make the substrate more convenient for reaction substrates to approach the active species. Thus, improving the utilization of TS‐1 active species and the total amount of active substance have become a focus of current research. Developing a TS‐1 catalyst with hierarchically structured channels, a high proportion of active species, low cost, and high stability is required for industrial applications.

A review of recent synthetic methods or approaches aimed at enhancing the activity of TS‐1 is presented. This paper briefly summarizes the catalytic performance of several Ti species in TS‐1 zeolite in reactions such as ammoximation, epoxidation, oxidative desulfurization, and other reactions, and discusses active titanium species and active intermediates. While the tetra‐coordinated framework Ti as an active species has been widely recognized, other coordinated Ti species even exhibit superior catalytic performance to tetra‐coordinated framework Ti in some oxidation reactions. However, there is currently a lack of effective and direct methods for synthesizing the desired coordinated Ti species. Wang et al.^[^ ^16^ ^]^ have provided a comprehensive and systematic exposition on the regulatory mechanisms of Ti species in TS‐1via a series of effective strategies, including measures to hinder the formation of extra‐framework Ti species, method to modulate the coordination state and local electronic environment of Ti, and strategies to optimize the distribution of Ti species within the framework. Li et al.^[^ ^27^ ^]^ reviewed TS‐1′s latest advances in propylene epoxidation (HPPO & gas‐phase routes), highlighting green preparation/modification strategies‐cost‐effective raw materials, novel synthesis pathways, enhanced mass transfer in crystals, and post‐modification performance boosts—for sustainable production.This paper briefly outlines the three stages in hydrothermal synthesis of zeolites: hydrolysis, crystallization, and post‐treatment. By regulating the hydrolysis process, the generation of anatase TiO_2_ is suppressed. Controlling the crystallization process enhances the framework Ti, reduces anatase TiO_2_, and yields highly active coordinated Ti species. During the post‐treatment phase, Ti species are redistributed by controlling dissolution and recrystallization, thereby introducing more Ti atoms into the TS‐1 zeolite framework. Through the discussion of the active species, reaction pathway and synthesis process of titanium silicalite molecular sieve, the synthesis strategy of high active catalyst are summarized, thereby providing guidance for the synthesis of high efficient titanium silicalite molecular sieve catalyst.

## 2 Applications and Reaction Pathways

Combined with H_2_O_2_, TS‐1 serves as a catalyst for the selective oxidation of numerous organic substrates, yielding minimal by‐products, such as the oxidation of hydrocarbones, the epoxidation of olefins, isomerization and ammoximation of ketone.^[^ ^27^, ^28^, ^29^, ^30^, ^31^ ^]^ The quantity, location, and condition of active species play a pivotal role in determining the catalytic activity of TS‐1 zeolite.

### 2.1 Intermediate Ti Peroxy Species

**Figure 3:**
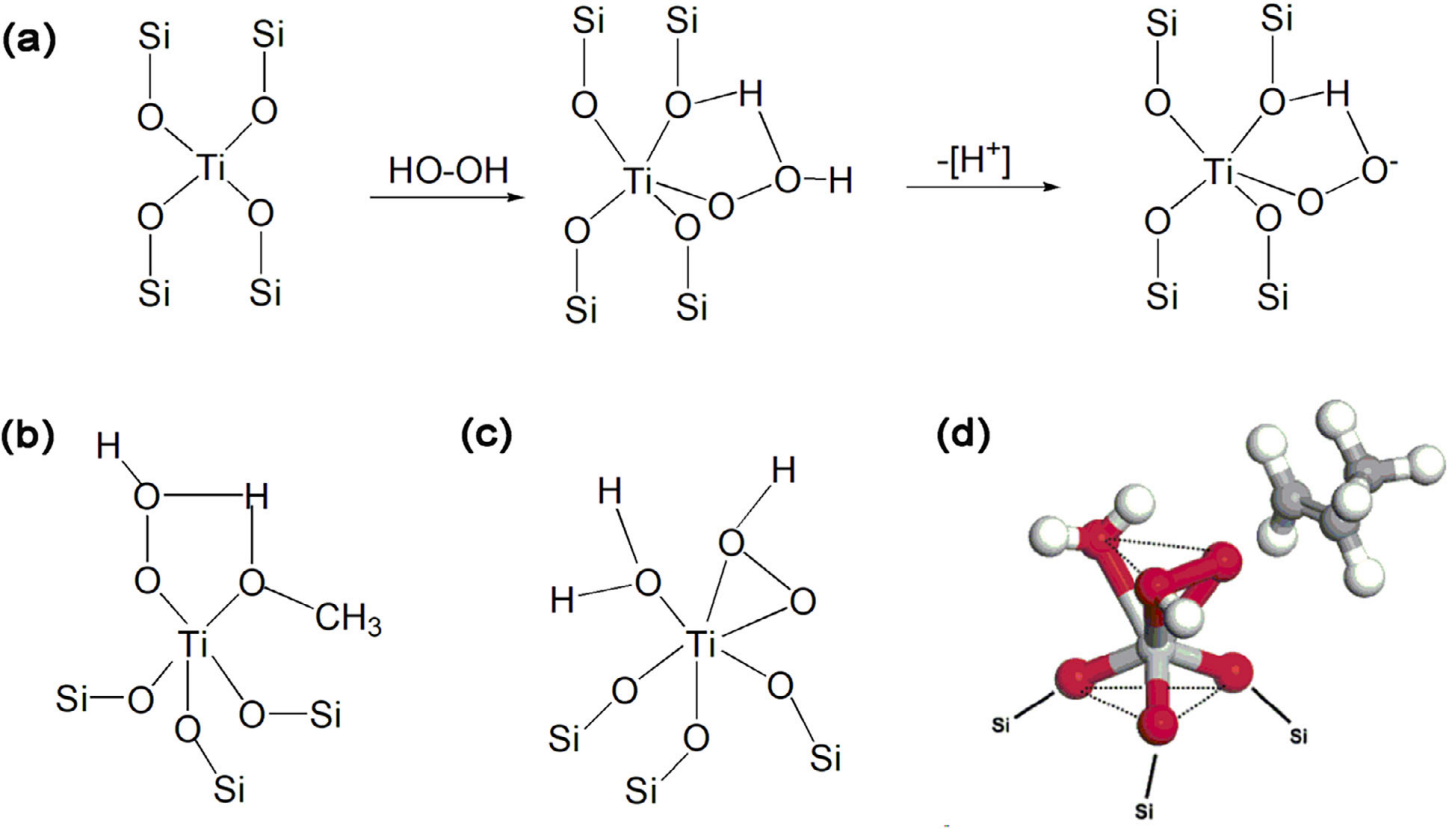
a) Five‐membered ring (5MR) intermediate Ti peroxy species formed by TS‐1 zeolite and H_2_O_2._ b) Transition state titanium of penta‐coordinated titanium with five member ring structure (5MR). c,d) Transition state titanium of hexa‐coordinated titanium with distorted octahedral structure. Reproduced with permission.^[^ ^31^ ^]^ Copyright 2004, American Chemical Society.

In the heterogeneous catalytic reaction, tetra‐coordinated Ti responsible for the catalytic activity can activate the reactant molecules and promote the reaction by reacting with hydrogen peroxide to form a five‐membered ring (5MR) intermediate Ti peroxy species (Ti‐OOH)^[^ ^29^, ^30^, ^31^ ^]^ (**Figure** **3**a). The debate on the oxidation mechanism of the tetra‐coordinated framework Ti has been widely recognized, but the discussion on the specific oxidation mechanisms of other coordinated titanium species is still incomplete, and there are also lack of effective and direct synthesis methods for the required coordinated titanium species.

To attain a more profound comprehension of the specific reaction mechanisms and active intermediates involved in TS‐1‐catalyzed reactions, researchers have leveraged various in situ characterization techniques to analyze active species during oxidation reactions. For instance, in 2001, Lin et al.^[^ ^32^ ^]^ first utilize in situ FT‐IR to directly observe the active oxidation in TS‐1 zeolite loaded with H_2_O_2_. The infrared absorption observed at 837 cm^−1^ and a broad band centered ≈3400 cm^−1^ indicated the generation of a stable Ti‐OOH (η^2^) species at room temperature. This Ti‐OOH species demonstrates the ability to oxidize propylene in the dark and directly oxidize ethylene under ligand‐to‐metal charge‐transfer chromophore excitation. Zhang et al.^[^ ^33^ ^]^ employ in situ diffuse reflectance fourier transform infrared spectroscopy (DRIFTS) to examine the generation of active intermediates (Ti‐OOH) during the liquid‐phase ammoximation of cyclohexanone. The peroxo Ti‐OOH species was found to react with imines to produce oximes. In situ DRIFTS^[^ ^34^ ^]^ can also be utilized to reveal the impact of the concentration of surface hydroxyl groups on the adsorption and conversion behavior of PO. The limited surface hydroxyl groups and acidic sites hinder the adsorption and subsequent conversion of the target product, PO. In contrast, the Ti(VI)‐L sample shows strong adsorption of PO, promoting its subsequent conversion into bidentate carbonaceous species. Additionally, ultraviolet resonance Raman (UV‐Raman) spectroscopy offers precise and sensitive discrimination between framework and extra‐framework Ti within TS‐1. To enhance the accuracy of Ti species structural analysis, in situ UV‐Raman is utilized to comprehensively study active Ti species and intermediates within TS‐1 zeolites, using propylene as a probe reactant.^[^ ^35^ ^]^ At different stages of in situ measurement, TiOOH (η^2^) species and triangular Ti(O_2_) species generated by physical adsorption of H_2_O_2_ can be observed. By leveraging a synergistic approach of in situ UV‐Raman spectroscopy and density functional theory (DFT) calculations, Li et al.^[^ ^19^ ^]^ unveiled that high‐coordination Ti species, including penta‐ and hexa‐coordinated Ti, could trigger side reactions such as propylene oxide (PO) ring‐opening. Xiong et al.^[^ ^36^ ^]^ utilized in situ UV‐Raman spectroscopy coupled with gas chromatography to investigate the propylene epoxidation process, and identify that a hexa‐coordinated TiOOH(η) active intermediate species containing a methanol molecule (or an H_2_O molecule) plays a pivotal role in the propylene epoxidation reaction. Yang et al.^[^ ^37^ ^]^ combined DFT calculations with in situ ^29^Si and ^31^P solid‐state nuclear magnetic resonance experiments to elucidate the distinct roles of perfect tetrahedral Ti(SiO)_4_ species and defective tetrahedral Ti(SiO)_3_OH species.

**Figure 4:**
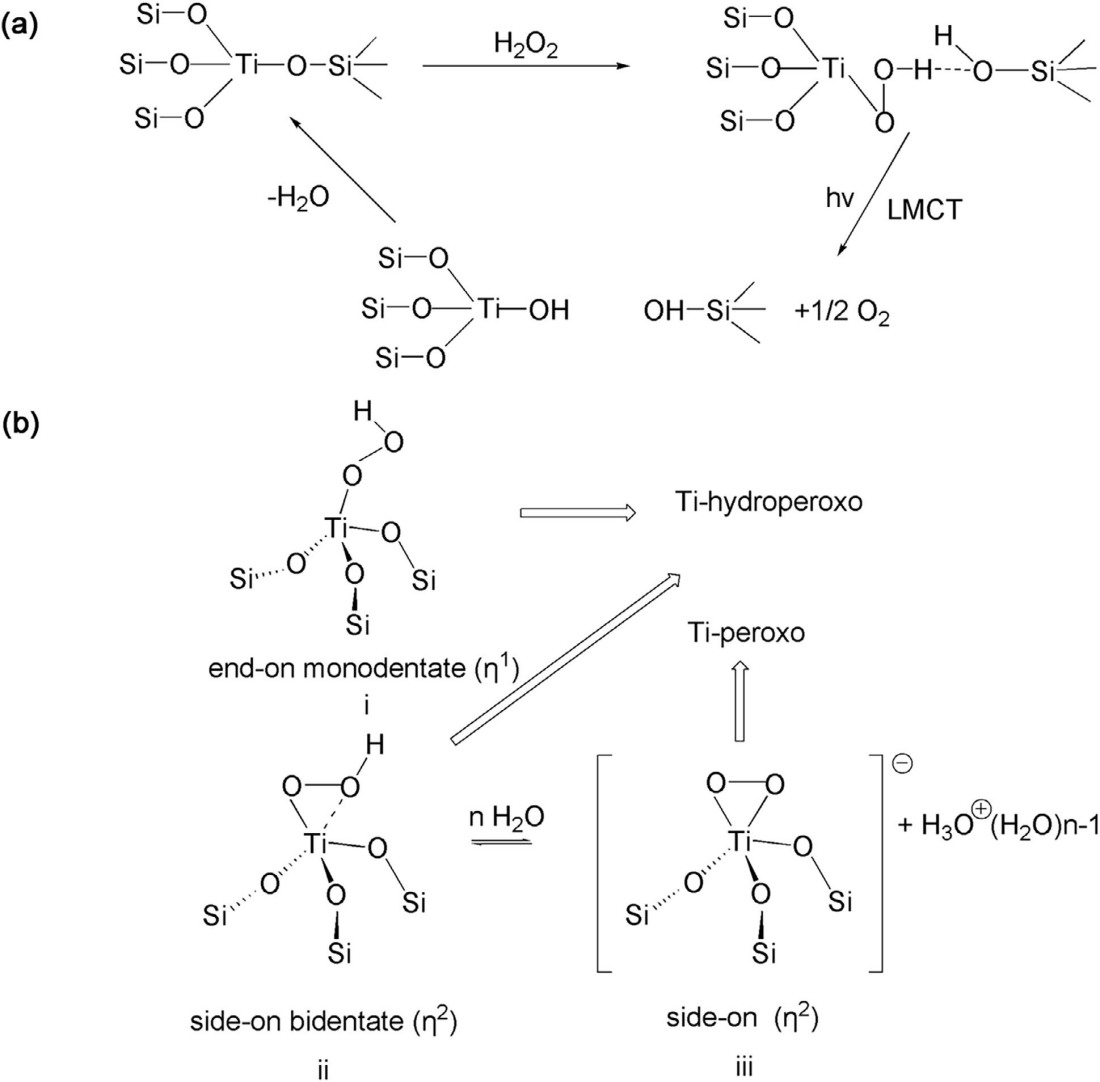
Possible formation process and intermediates from in situ technique: a) TiOOH (η^1^) species. b) Ti‐hydroperoxo and Ti‐ peroxo from TiOOH (η^1^ and η^2^) and Triangular Ti(O_2_) species.

Other type penta‐coordinated and hexa‐coordinated intermediate Ti peroxy species derive from isolated four coordinated titanium species under reaction conditions have been reported to be generated by the expansion of the tetra‐coordinated Ti surface when it comes into contact with H_2_O, H_2_O_2_, reactants, and solvent molecules. The penta‐coordinated transition state titanium with five member ring structure (5MR) can be formed by tetra‐coordinated Ti with H_2_O_2_ and ROH (Figure 3a,b), and further transformed into a 5MR Ti peroxy species (Ti‐OOH).^[^ ^29^, ^38^ ^]^ The hexa‐coordinated transition state titanium with distorted octahedral structure, contain three Ti─O─Si bonds, one adsorbed water and two oxygens from the H_2_O_2_ (Figure 3c,d).^[^ ^31^ ^]^ As reaction intermediate, these penta‐ and hexa‐ coordinated transition state Ti species are deemed more reactive in oxidative reactions involving liquid‐phase H_2_O_2_. The process formation process and intermediates of TiOOH (η^1^ and η^2^) species and triangular Ti(O_2_) species (**Figure** **4**a,b) can be captured by in situ technique.^[^ ^30^, ^35^ ^]^ And the highly reactivity of TiOOH (η^1^ and η^2^) species and triangular Ti(O_2_) species lead to their quick to Ti hydroperoxides and Ti peroxides.

Other coordinated Ti species within TS‐1 may also be active in the oxidation process. Based on the research on the catalytic oxidation process utilizing TS‐1 /H_2_O_2_, it can be seen that the core of the catalytic performance is the intrinsic catalytic oxidation ability of Ti species, although the reaction performance is affected by reaction conditions and pore diffusion restrictions. An in‐depth of the existence form of titanium species and the reaction mechanism is needed to further improve the catalytic activity of oxidation reaction.

### 2.2 Oximation

**Figure 5:**
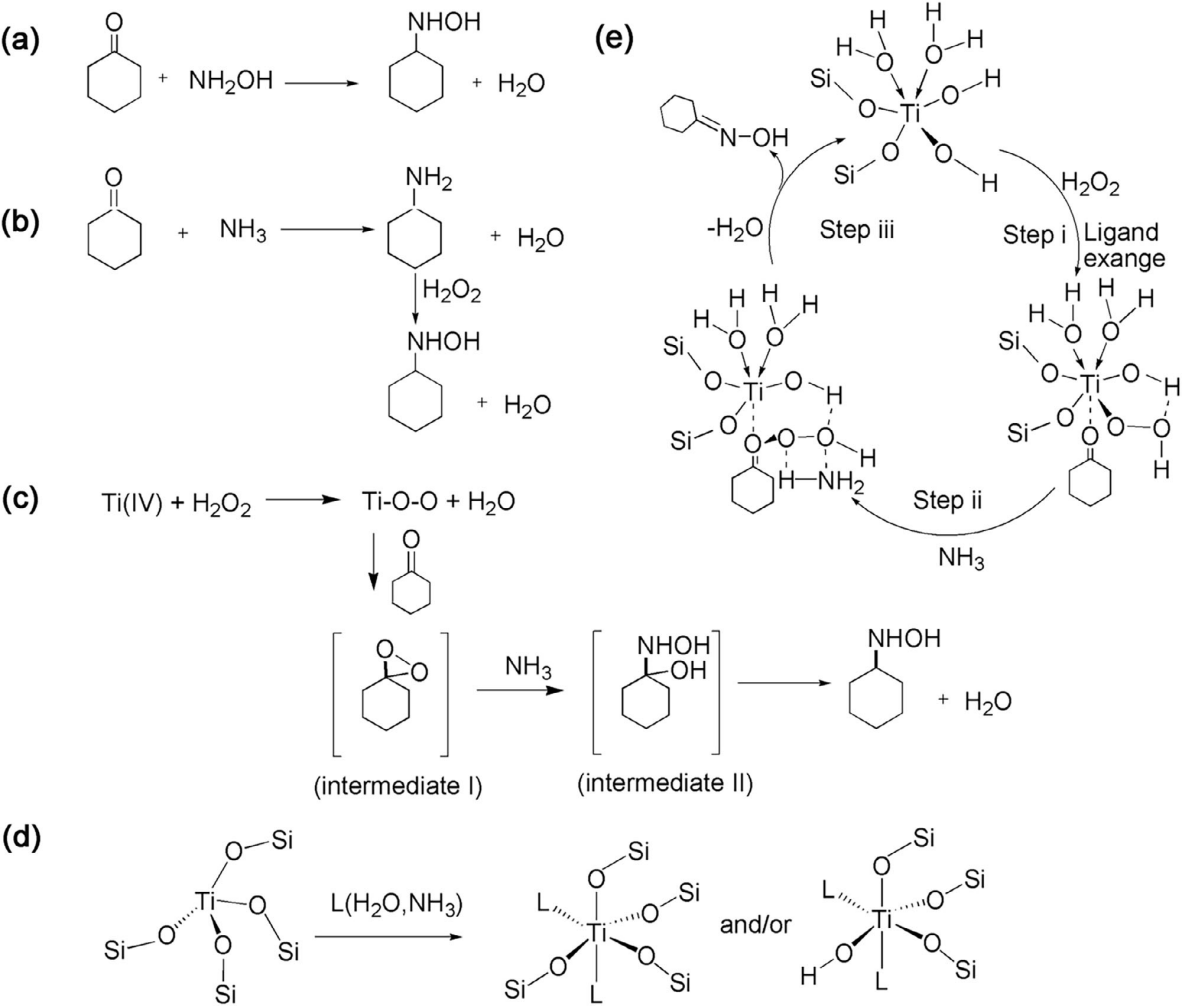
Reaction mechanism of TS‐1/H_2_O_2_/NH_3_ system: a) Imine route. b) Hydroxylamine route. c) Proposed reaction mechanism of cyclohexanone oxime formation. d) Adsorption of L(NH_3_ or H_2_O) on TS‐1 and the formation of hexa‐coordinated Ti L_2_(OSi)_4_. e) Possible reaction pathways of cyclohexanone oxidation pathway.

In the 1980s, the TS‐1 catalyst was introduced for synthesizing oxime^[^ ^39^ ^]^ under mild conditions with ammonia and H_2_O_2_. Since then, TS‐1 has achieved remarkable success in green synthesis, especially for nitrogen‐ and oxygen‐containing organic intermediates, employing H_2_O_2_ as oxidant.^[^ ^40^, ^41^, ^42^ ^]^ The TS‐1/H_2_O_2_ system has been extensively applied in ketone ammoximation, demonstrating outstanding performance.^[^ ^43^ ^]^ Ample evidence supports the coordination of NH_3_ and H_2_O molecules to tetrahedral Ti^4+^, leading to the expansion of their coordination intermediates. The adsorption of NH_3_ on TS‐1 reveals that small molecule ligands like NH_3_ and H_2_O can be inserted into the coordination spheres of the framework Ti (Ti(OSi)_4_) to form hexa‐coordinated Ti species such as (SiO)_4_TiL_2_, and/or (SiO)_3_Ti(OH)L and present distorted octahedral structures (**Figure** **5**d).^[^ ^44^ ^]^

The reaction mechanism of TS‐1/H_2_O_2_ system has always been a concern of researchers. There are two main reaction pathways for oximation of TS‐1/H_2_O_2_/NH_3_ system: hydroxylamine pathway (Figure 5a) and imine pathway (Figure 5b). The hydroxylamine pathway suggests that NH_3_ is first oxidized to hydroxylamine by H_2_O_2_ on the framework Ti. Then, hydroxylamine reacts with substrate ketone undergoing a non‐catalytic process to obtain the product target product oxime.^[^ ^45^, ^46^ ^]^ Lots of experimental and calculating approach have provided evidence that Ti‐OOH serve as the oxidizing agent and the existence of hydroxylamine in the TS‐1/H_2_O_2_ system.^[^ ^46^ ^]^ Therefore, the hydroxylamine mechanism has gained widely recognized.

The imine pathway suggests that the Ti‐OOH intermediate formed by the tetra‐coordinated Ti and H_2_O_2_ molecule. NH_3_ exhibits a preference for reacting with substrate ketone, yielding the unstable intermediate imine. This intermediate is subsequently oxidized to cyclohexanone oxime by Ti‐OOH intermediate.^[^ ^47^, ^48^ ^]^ The role of titanium would be to activate the oxygen and then insert on the imine intermediate.^[^ ^49^ ^]^ Following the treatment of TS‐1with H_2_O_2_, the presence of peroxytitanium species Ti‐OOH has been verified and indicated the reaction from imine to produce oxime. Although the mechanism of imine can give some explanation for the existence of the byproduct dicyclohexylamine peroxide, in the presence of H_2_O, imine is very unstable, and the existence of imine has not been effectively confirmed in the actual reaction, so the mechanism has been controversial.

In addition, different mechanisms have also been put forward as well, including the oxidation pathway of cyclohexanone (Figure 5c).^[^ ^49^ ^]^ H_2_O_2_ undergoes adsorption onto the TS‐1 catalyst, leading to the formation of a complex peroxo‐titanium species. Ti‐O‐O species can oxidize cyclohexanone substrates to form peroxide intermediates I. These intermediates can then react with NH_3_ to yield peroxide intermediates II. Subsequently, cyclohexanone oxime is formed by losing one water molecule.

The traditional methods of acetaldoxime synthesis are via hydroxylamine pathway and acid catalyzed oxidation. Acetaldehyde ammoximation^[^ ^50^ ^]^ is conducted with TS‐1 as catalyst and H_2_O_2_ as oxidant, NH_3_ is first oxidized to NH_2_OH by TS‐1/H_2_O_2_, then, NH_2_OH reacts with CH_3_CHO forming CH_3_CH = NOH. Under the action of tetra‐coordinated framework Ti, liquid‐phase ammoximation reaction in TS‐1/H_2_O_2_ system shows high conversion and oxime selectivity. The liquid‐phase ammoximation in TS‐1/H_2_O_2_ system show high conversion and oxime selectivity.^[^ ^51^, ^52^ ^]^

Furfural oxime (FO) is formed by the liquid phase ammoximation of furfural with NH_3_ and H_2_O_2_ catalyzed by TS‐1 molecular sieve.^[^ ^41^ ^]^ Different products are obtained in the furfural ammoximation via the imine route and hydroxylamine route, respectively. Furfural is catalysed to FO by ammoximation via hydroxylamine route, and furfural is non‐catalysed to 2‐furamide and 2‐furoic acid via imine route. The results shows the coexistence of FO with 2‐furamide and 2‐furoic acid, indicating that the imine and the hydroxylamine route occurred simultaneously or competed with each other.

Another hydroxylamine mechanism^[^ ^53^ ^]^ (Figure 5e) dominated by defective tetra‐coordinated Ti (SiO_4_)_2_(OH)_2_ as the active species suggests that it become hexa‐coordinated Ti species, such as Ti(OH)(H_2_O)_2_(SiO_4_)_3_ and Ti(OH)(NH_3_)_2_(SiO_4_)_3_ in the presence of H_2_O_2_, water, and ammonia. Subsequently, H_2_O_2_ undergoes substantial activation, leading to the generation of TiOOH species. These species then reacts with NH_3_ molecules to produce hydroxylamine. Cyclohexanone promptly interacts with hydroxylamine, resulting in the formation of cyclohexanone oxime.

TS‐1 exhibits excellent performance in ammoximation reaction, and its possible mechanism has been extensively studied. It is evident that there is an inherent link between Ti active species in TS‐1 molecular sieve and catalytic efficiency. That is to say, further research is needed to investigate mechanism formed on TS‐1 catalyst in ammonia and hydrogen peroxide, as well as Ti species types in the MFI framework. Understanding these issues will be favor to develop efficient TS‐1 catalysts and methods for the conversion of ketones into corresponding oximes.

### 2.3 Epoxidation

**Figure 6:**
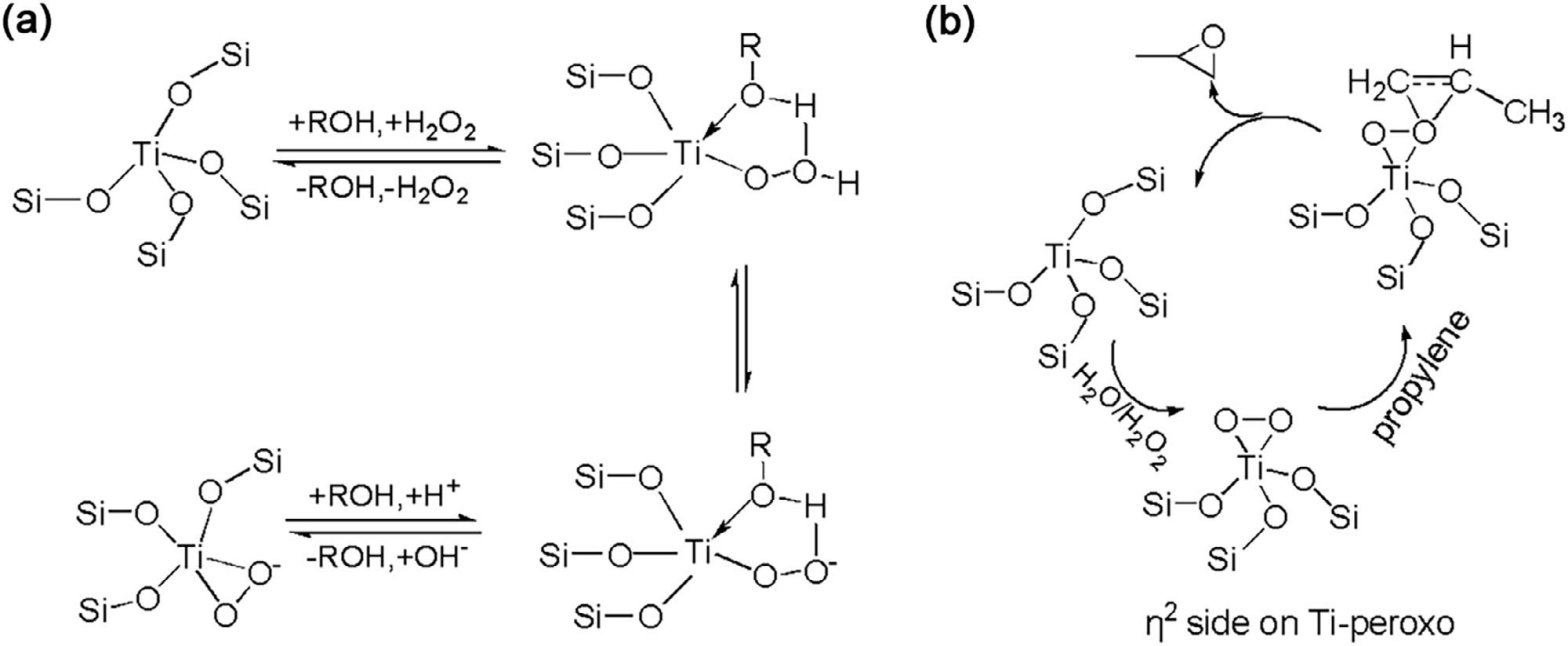
a) Ti‐O‐O five‐membered ring intermediate. b) Proposed Ti‐peroxo species η^2^ side‐on Ti‐peroxo (Ti‐O‐O) intermediate by tetra‐coordinated Ti and H_2_O_2_ in epoxidation mechanism catalyzed by TS‐1.

The hydrogen‐peroxide‐to‐propylene‐oxide (HPPO) process is an economically and environmentally benign process.^[^ ^54^, ^55^ ^]^ HPPO process employs H_2_O_2_ as oxidant to catalyze olefin epoxidation, and the by‐product is only water. TS‐1 demonstrates high catalytic activity and stability in this process under mild reaction conditions.^[^ ^56^, ^57^, ^58^, ^59^ ^]^ After proper treatment with TPAOH, mesopores and hexa‐coordinated Ti are generated in TS‐1 crystalls through a dissolution‐recrystallization process, and this TPAOH‐treated TS‐1 has been utilized in the industrial HPPO process. Compared to microporous TS‐1 without TPAOH treatment, the TPAOH‐treated TS‐1 demonstrates superior catalytic performance. The HPPO process catalyzed by TS‐1 treated by TPAOH has been successfully operated in an industrial plant for over 6000 h, maintaining nearly the same catalytic activity as initially observed. Under a range of operating conditions, the TOF of H_2_O_2_ is ≈31–32 mmol g^−1^h^−1^ (X(H_2_O_2_) is 96–99%), while the selectivity for PO is also 96–99%.^[^ ^56^ ^]^ The reaction mechanism for propylene epoxidation remains open. The most widely recognized epoxidation pathway is the five membered ring (5MR) mechanism (**Figure** **6**a). The 5MR mechanism suggests that the tetra‐coordinated TiO_4_ first interact with H_2_O_2_, leading to the formation of the active intermediate Ti–O−O−H, then solvent (alcohol or water) coordinates with titanium and forms a five‐membered ring intermediate. The oxygen atom in the 5MR intermediate directly connects to titanium atoms readily attacks the olefin double bond, effectively transferring active oxygen to achieve epoxidation.^[^ ^60^ ^]^ In this proposed mechanism, the penta‐coordinated Ti intermediate plays a pivotal role in facilitating the oxidation reactions. When methanol, a protic solvent, is introduced into the TS‐1/H_2_O_2_ system, it is noted that methanol solvent exerts an influence on the propylene epoxidation process. Owing to the formation of hydrogen bonds, the co‐adsorption of CH_3_OH and H_2_O_2_ is notably enhanced. More significantly, as CH_3_OH is involved in the hydrogen transfer process and the formation of a hydrogen bond network, the energy barrier for H_2_O_2_ dissociation can be markedly reduced ≈13 kcal mol^−1^, consequently altering the rate‐limiting step.^[^ ^61^ ^]^ The solvent directly affects the formation, stability, and transfer of the Ti‐OOH active intermediate in the reaction pathway.

The catalytic activity of TS‐1 is mainly attributed to active TiO_4_ species within the zeolite framework. These species interact with H_2_O_2_ to generate active Ti‐peroxo species.^[^ ^36^, ^61^ ^]^ Another Ti‐peroxo mechanism suggests (Figure 6b) that the tetra‐coordinated TiO_4_ initially interact with H_2_O_2_/H_2_O to form η^2^ side‐on Ti‐peroxo (Ti‐O‐O) intermediate species. These intermediates then directly oxidize the olefin molecules to accomplish the epoxidation of olefins.^[^ ^36^ ^]^ Furthermore, Raman spectrum provides evidence for the formation of Ti OOH (η^2^) intermediate in the H_2_O_2_/H_2_O/CH_3_OH system, and this intermediate subsequently undergoes an oxidation reaction with propylene, leading to the production of epoxypropane. Non‐framework Ti species (amorphous TiO_2_ and anatase TiO_2_) promote ring opening reactions of PO to generate PG/PGME.The above results confirm that framework Ti are the active species for the production of PO, and the epoxidation of propylene to PO proceeds via a Ti–OOH (η^2^) intermediate.^[^ ^19^ ^]^

**Figure 7:**
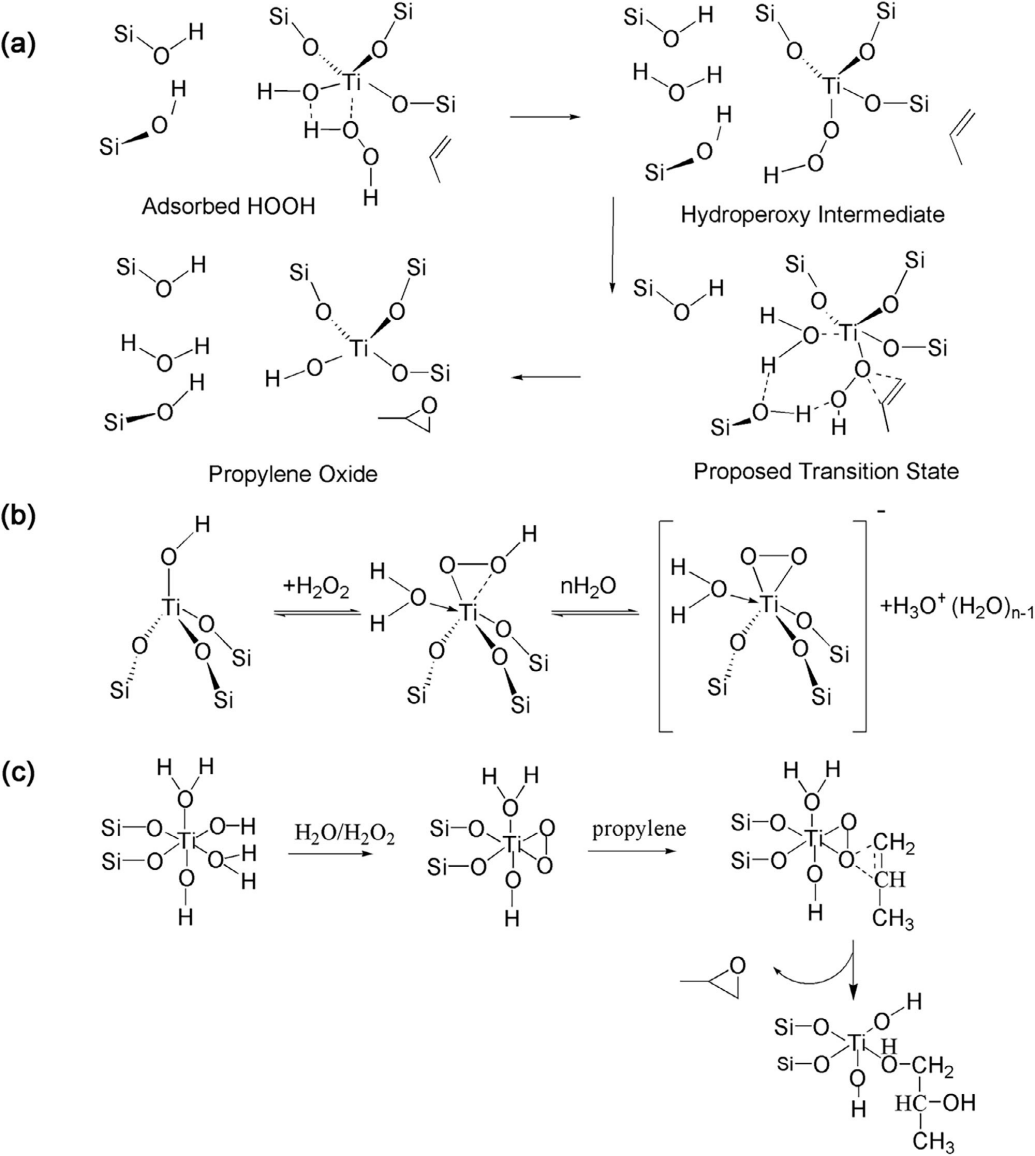
Proposed pathway for HOOH/propylene epoxidation to PO on Ti active species. a,b) Pefective tetra‐coordinated Ti (Ti(OH)(OSi)_3_). c) Hexa‐coordinatied Ti (mononuclear TiO_6_, Ti(OH)_2_(H_2_O)_2_(OSi)_2_) species in TS‐1.

Compared with Ti(OSi)_4_, Ti(OSi)_3_OH species can react with H_2_O_2_ easily to form more stable Ti‐ηOOH active intermediate structure. As an active intermediate, activation energy required for the formation of five‐membered ring between open Ti(OSi)_3_OH and H_2_O_2_ is lower than that of Ti(OSi)_4_. Therefore, in the olefin epoxidation reaction, open Ti(OSi)_3_OH often shows better catalytic performance in the catalytic reaction.^[^ ^62^ ^]^ The content of Si‐OH around the active center in TS‐1 have a significant influence the catalytic activity of the molecular sieve. Increasing the quantity of Si‐OH groups around the active Ti center can strengthen hydrogen bond interactions with water molecules. Through interactions with active intermediates and polar surfaces, this enhancement facilitates the transfer process of active oxygen (O), thereby improving the reaction performance in oxidation reaction. Wells and colleagues^[^ ^31^ ^]^ discover a new epoxidation pathway about Ti(OSi)_3_OH active species through DFT calculations (as shown in **Figure** **7**a). In the epoxidation route: 1) H_2_O_2_ inserts into the Ti (OSi)_3_OH, and the terminal Ti hydroxyl group undergoes hydrolysis to yield to form an active hydroperoxy intermediate Ti(OSi)_3_OOH. 2) A seven‐membered ring intermediate is formed through the interaction of H_2_O, adjacent Si‐OH and propylene near Ti site. 3) Near the active center, oxygen undergoes abstraction, facilitating the formation of propylene oxide that subsequently detaches from the surface of the TS‐1 catalyst. In situ UV Raman spectroscopy captures physisorbed H_2_O_2_ and triangular Ti(O_2_) species at various stages in propylene epoxidation. The results obtained by GC‐Raman reveal that seven‐membered ring Ti–OOH (η^2^) are crucial active intermediate in methanol‐included propylene epoxidation reaction process, contributing to enhanced stability and superior catalytic activity.^[^ ^31^ ^]^ Defective Ti(OSi)_3_(OH) represents a highly active oxidizing species. As illustrated in Figure 7b, defective Ti species can transform into a hexa‐coordinated Ti−OOH (η^2^) complex under the influence of H_2_O_2_. Furthermore, under the impact of additional water molecules, an equilibrium exists between the hexa‐coordinated Ti−H (η^2^) complex and its corresponding hexa‐coordinated η^2^ Ti‐peroxo complex.^[^ ^35^ ^]^

With the deepening of research, researchers have also discovered hexa‐coordinated and penta‐coordinatied Ti species, and these species are considered more active than tetrahedral in HPPO.^[^ ^63^ ^]^ The pathway for propylene epoxidation to PO have been reported on the hexa‐coordinatied Ti (mononuclear TiO_6_, Ti(OH)_2_(H_2_O)_2_(OSi)_2_) species within TS‐1 (Figure 7c). It has been proposed that similar Ti‐peroxo species were formed on both the “TiO_6_” and tetra‐coordinated titanium species when in contact with H_2_O_2_/H_2_O solution. Following interaction with propylene at 60 °C, the η^2^ side‐on Ti‐peroxo species facilitate the oxidation of propylene, leading to the formation of PO, as the final product. This result shows that the framework TiO_4_ and TiO_6_ species are also highly active in propylene epoxidation. But Highly coordinated Ti species have greater acidity due to more terminal Ti‐OH or Si‐OH groups, a characteristic that subsequently diminishes the selectivity of propylene oxide.

TS‐1 catalyst can also be applied to the epoxidation of other olefins such as ethylene, 1,2‐butylene, cyclohexene and cyclopentene,^[^ ^64^, ^65^ ^]^ and the mono epoxidation of diene (allyl ether, acrylate and allyl methacrylate, and 1,3‐butadiene).^[^ ^66^, ^67^ ^]^ The framework TiO_4_ species in TS‐1 are still considered the main active species. The relation between Ti species and activity of TS‐1 in HPPO reaction were investigated.^[^ ^68^ ^]^ The outcomes indicate that the activity of catalysts are linear correlation with the content of framework Ti. As the amount of non‐framework Ti species rises, there is a marginal decline in the selectivity of PO, accompanied by a slight increase in the conversion of H_2_O_2_.^[^ ^69^, ^70^ ^]^

**Figure 8:**
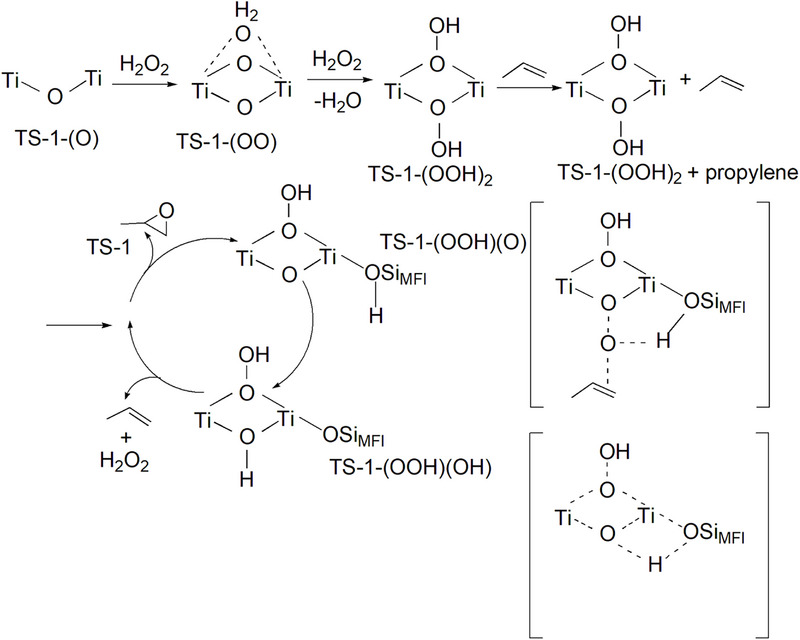
Bridging peroxo species on dinuclear titanium sites with H_2_O_2_.

Recently, Gordon et al.^[^ ^26^ ^]^ proposed that the binuclear Ti in TS‐1 is mainly responsible for the activity of propylene epoxidation reaction through solid ^17^O NMR technology and theoretical calculation. It has been found the bridging peroxy species formed on the binuclear titanium site with H_2_O_2_ played a key role in propylene epoxidation (**Figure** **8**). Moreover, DFT calculations reveal that the synergistic interaction between two titanium atoms facilitates propylene epoxidation through a low‐energy reaction route, featuring a crucial oxygen‐transfer transition state analogous to that observed in olefin epoxidation mediated by peracids. The dinuclear Ti in TS‐1 is proposed to contribute higher efficiency than traditional tetra‐coordinated framework Ti.

### 2.4 Other Application

Hydroxylation of phenol with H_2_O_2_ over TS‐1 is an environmental friendly route. Tetra‐ coordinated Ti is considered as the active species for this hydroxylation process.^[^ ^71^ ^]^ Nano‐sized TS‐1 demonstrates superior anti‐coking properties and enhanced selectivity toward phenyl acetaldehyde during the isomerization of styrene oxide to phenyl acetaldehyde.^[^ ^25^ ^]^ A modified MFI type zeolite with balanced acidity and pore architecture may prove advantageous for this reaction. The utilization of TS‐1 as a photocatalyst has garnered significant interest.^[^ ^72^ ^]^ In photocatalytic systems, the TiO_4_ species [Ti^4+^‐OL^2−^] on the catalyst surface undergo photoexcitation, forming a charge‐transfer excited state [Ti^3+^‐OL^−^]*, and this excited state then serves as the active species. Photoactivation of 1,3,5‐trihydroxybenzene show particularly high conversion rates (12‐21%) on TS‐1. This catalytic property is attributed to the TiO_4_ active species and the well‐structured pore system of the catalysts.

**Figure 9:**
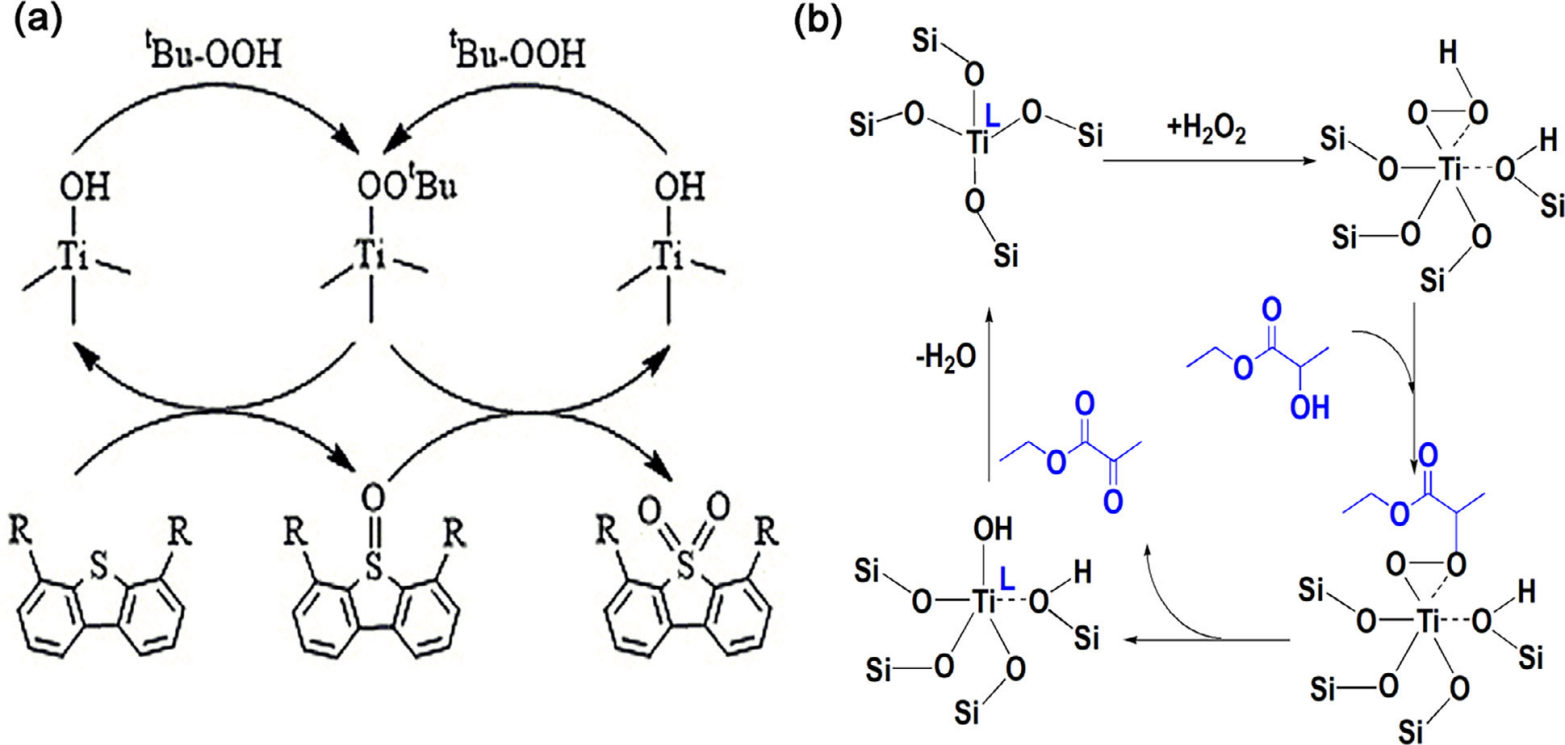
a) Proposed mechanism for ODS process. Reproduced with permission.^[^ ^73^ ^]^Copyright 2022, The Author(s). Published by Tech Science Press. b) Proposed mechanism for oxidation of ethyl lactate to ethyl pyruvate.

TS‐1 catalyst is widely used for oxidation desulfurization (ODS) under mild conditions with framework tetra‐coordinated Ti as active species.^[^ ^72^, ^73^, ^74^, ^75^, ^76^ ^]^ The open tetra‐coordinated Ti(OH)(OSi)_3_ species within TS‐1 catalyst serves as the principal active center, playing a crucial role in facilitating the ODS processes of dibenzothiophene (DBT) and 4,6‐dimethyldibenzothiophene (4,6‐DMDBT) (**Figure** **9**a). The active intermediate TiOOt Bu is formed by the reaction of tert‐Butyl hydroperoxide (TBHP) on the Ti(OH)(OSi)_3_. The nucleophilic attack occurs through the reaction of sulfur atoms in DBT (or 4,6‐DMDBT) with TiOOt Bu species to form sulfoxides. These sulfoxides exhibit high instability and are promptly oxidized to sulfone by the Ti OOt Bu species.^[^ ^73^ ^]^

TS‐1 catalyst are catalytic active in various selective oxidation process.^[^ ^74^ ^]^ TS‐1 can effectively utilize renewable biomass.^[^ ^75^, ^76^ ^]^ Under microwave conditions, TS‐1 efficiently facilitates the rapid conversion of lignin into succinic acid and malic acid, with maximum yields of 11.3% and 19.5%, respectively.^[^ ^75^ ^]^ The framework tetra‐coordinated Ti species (Ti(OSi)_4_) in TS‐1 zeolite reacts with H_2_O_2_ to yield Ti(η^2^OOH). This intermediate undergoes a dehydration reaction with the hydroxyl group of ethyl lactate, leading to the formation of ethyl pyruvate (Figure 9b).^[^ ^77^ ^]^ TS‐1 exhibits excellent stability and can catalyze the one‐step alcoholysis of fructose to produce methyl lactate with stable cycling performance over 14 cycles.^[^ ^78^ ^]^ Moreover, the novel TS‐1/C_3_N_4_ composite material serves as an effective photocatalyst for the degradation of antibiotic wastewater containing erythromycin and ofloxacin, demonstrating superior catalytic activity.^[^ ^79^ ^]^

Small crystal size, b orientation and a large average pore diameter can reduce internal diffusion limit, enabling reaction substrates to readily access the active centers.^[^ ^80^, ^81^ ^]^ The significant role of Ti species in various catalytic reactions within the TS‐1 catalyst is undeniable, but different Ti species have different catalytic capabilities for substrates. The tetra‐coordinated Ti is usually identified to be active species for a series of selective oxidation of organics. The penta‐, hexa‐coordinated Ti species, defect Ti species could also act as active species to form the active intermediate Ti‐peroxo species.^[^ ^82^ ^]^ Anatase TiO_2_ crystals have been observed to reduce the efficiency of H_2_O_2_ utilization and encourage excessive product conversion, a situation that should be minimized or avoided whenever feasible.

The micro‐environment structure surrounding the active center significantly exerts a substantial influence on both the catalytic mechanism of TS‐1/H_2_O_2_ system and the formation of active intermediates. Specifically, in the ammonoximation reaction system, the alkaline environment tends to erode the silica framework of TS‐1, resulting in the elimination of framework TiO_4_. Solvent plays a crucial role in shaping the reaction mechanism by involving hydrogen transfer process and the formation of a hydrogen bond. The charge state of Ti species determines their varying Lewis acidity, a critical factor in activating H_2_O_2_. Enhancing the positive charge on Ti active sites can improve their catalytic activity. Additionally, the content of Si‐OH groups around Ti active centers modify the reaction pathway, leading to the formation of different active intermediates. The increase of Si‐OH content promotes the enrichment of H_2_O_2_, thereby enhancing its activity in oxidation reaction.

The coordination state of Ti in TS‐1 have a significant influence the catalytic activity of the molecular sieve. It has been discovered that the tetra‐coordinated Ti species, Ti(OSi)_4_ or Ti(OSi)_3_(OH), can directly react with H_2_O_2_/H_2_O to generate various types of Ti peroxide species, including the Ti‐OOH 5‐MR, Ti‐(η^2^‐OO)‐3MR, and Ti(OSi)_3_OOH. Additionally, tetra‐coordinated Ti species can adsorb ligands (NH_3_, H_2_O, H_2_O_2_, et al.) to form high‐coordinated Ti active species. The high‐coordinated Ti species (penta‐ and hexa‐coordinated) exhibit a greater tendency to resemble similar Ti peroxide species in the presence of H_2_O_2_/H_2_O. The dinuclear Ti species can combine with H_2_O_2_/H_2_O to yield a bridging peroxo species.

Although numerous studies have focused on the reaction mechanisms involving diverse Ti species, it is still widely acknowledged that tera‐coordinated Ti represents the predominant active species. This species is responsible for generating active intermediate peroxide species and engaging in the catalytic cycle alongside with H_2_O_2_. Furthermore, the presence of high‐coordinated Ti can significantly enhance the catalytic performance of TS‐1 in oxidation reactions.

Reaction mechanisms of tera‐, penta‐, hexa‐coordinate Ti, and dinuclear Ti vary in different oxidation processes, all of them are capable of forming various types of active intermediate titanium peroxide species when reacting with H2O2, thereby exerting a catalytic effect in a range of oxidation reactions. Although we have gained a certain level of understanding regarding the catalytic mechanisms exhibited by various active titanium species in selective oxidation reactions such as oximation and epoxidation, the specific details of these mechanisms and the precise structures of intermediate Ti peroxy species remain unclear. Furthermore, there is currently no definitive conclusion on which type of titanium active species is more suitable for which category of catalytic reactions. With the continuous advancement of in situ characterization techniques and theoretical calculation methods, the catalytic mechanisms of active titanium species in various oxidation reactions are being continuously deepened and refined. Given this, it is necessary for us to leverage more advanced in situ characterization tools and density functional theory calculations to further explore and establish more accurate catalytic mechanisms. This will enable us to more precisely select the appropriate types of active titanium species for oxidation reactions and thereby propel the application of TS‐1 molecular sieves in the catalytic field to new heights.

## 3 Approaches to Improving Active Center

TS‐1 molecular sieve exhibits remarkable catalytic efficacy in many selective oxidation reactions. The catalytic oxidation performance of TS‐1 is actually influenced by the microenvironment surrounding its Ti active centers, as this environment directly influences the formation, stability, and transfer of active intermediates. In the TS‐1/H_2_O_2_ catalytic system, solvents have a substantial impact on both the reaction activity and selectivity of productions. Additionally, the charge state of Ti species represents its inherent catalytic ability, with an increased positive charge at the Ti active site proving advantageous for boosting its catalytic performance.

The number, distribution and coordination state of Ti active species within TS‐1 molecular sieve determine its catalytic activity. Tuning the coordination state and charge state of Ti active species enables the improvement of intrinsic catalytic capability of TS‐1 zeolite.

The widely recognized active species are the framework tetra‐coordinated Ti and highly coordinated Ti within TS‐1 crystals. Therefore, the core of designing high‐efficient catalysts is to optimize the Ti distribution within the zeolite, increase the proportion of framework tetra‐coordinated Ti, and achieve the coexistence of high‐coordinated Ti species. The pore architecture of TS‐1 also determines its catalytic performance, because the mesoporous structure can improve the accessibility between substrate molecules and active Ti species, thereby improving the catalytic reaction capacity of the catalyst. Therefore, TS‐1 with hierarchical pore structure or layered structure is more prone to diffusion of substrate molecules in the mesoporous channel and high outer surface.

Due to the poor Ti content in the framework, many researchers are committed to increase the proportion of tetra‐coordinated Ti species within it. The hydrolysis and crystallization rates of silicon and titanium sources during TS‐1 synthesis process significantly impact the content, type of Ti incorporated into the TS‐1 crystals. Controlling both the synthesis and post‐treatment process is an effective means increasing Ti content in TS‐1. This study is provided on the synthesis of TS‐1 zeolite from the controlling the hydrolysis, crystallization, and the post‐treatment steps. The strategy about preparation and regulation of TS‐1 zeolite with rich framework Ti, high catalytic activity and accessibility of Ti species are reviewed. These studies will provide suggestions and guidance for understanding and preparing titanium silicate zeolite catalysts with high catalytic performance.

### 3.1 Hydrolysis Rate Matching Strategy

It is well known that the preparation of sol–gel precursor is one of the important processes in the synthesis of TS‐1 zeolite. During this process, the hydrolysis rate of Ti precursor significantly outpaces than that of Si precursor, resulting in a propensity for Ti monomers to undergo oligomerization. The mismatch in hydrolysis rate between the silicon and titanium sources is one of main factors leading to the generation of anatase TiO_2_ and other inactive titanium species.

#### 3.1.1 Slowing Down Hydrolysis of Ti Precursors

During the traditional hydrothermal synthesis of TS‐1, a moderate degree of hydrolysis of Si sources play a crucial role in effectively curbing the formation of extra‐framework Ti and enhancing the content of framework Ti. However, hydrolysis times that are either too short or too long impede the incorporation of titanium into the framework, thereby diminishing the catalytic performance of TS‐1 zeolites.^[^ ^83^ ^]^ A strategy is employed to slow down the hydrolysis of the Ti precursor, ensuring that its hydrolysis rate aligns with that of Si during the synthesis process.This approach effectively prevents the formation of Ti oligomers.

Previous studies have proposed various strategies to tackle this issue: 1) pH regulation of the precursor synthesis system. Fan et al.^[^ ^84^ ^]^ reduced the precursor's pH by adding (NH_4_)_2_CO_3_, slowing the Ti source's hydrolysis rate. This yields anatase‐free TS‐1 after 3–6 days of hydrothermal reaction; 2) Incorporation of organic mediating agents. Adding Triton X‐100^[^ ^85^ ^]^ to the precursor solution also produced anatase‐free TS‐1. Hydrogen peroxide, isopropanol, and Tween‐20 have been applied in hydrolysis process to inhibit the hydrolysis of Ti precursors to some extent by coordinating with Ti source.^[^ ^86^ ^]^ 3) Titanium source modification. Utilizing lactate titanium ammonium salt chelate^[^ ^87^ ^]^ as the titanium source, a bio‐phenolic solution derived from a plant extract is introduced as an adjuvant to regulate the hydrolysis rate of Ti, thereby aligning it with the crystallization rate. These measures can to some extent prevent the hydrolysis of Ti precursors, thereby reducing the content of TiO_2_. However, these methods are still difficult to achieve TS‐1 molecular sieves with abundant framework titanium species.

#### 3.1.2 Accelerating Hydrolysis of Si Precursors

During the TS‐1 synthesis process, in order to facilitate the incorporation of more Ti into the framework as framework Ti, the hydrolysis rate of Si precursor should be increased to align with that of Ti precursor. This adjustment is instrumental in preventing the formation of Ti oligomers. Therefore, adopting a strategy aimed at accelerating the hydrolysis rate of the Si precursor proves advantageous for the production of TS‐1 containing abundant framework Ti species.

Previous research has proposed various strategies to tackle this challenge: (1) pH regulation of the precursor synthesis system. TS‐1 zeolite featuring uniformly distributed framework titanium is successfully synthesized under high alkaline conditions, and demonstrates superior catalytic performance in both olefin and alkane oxidation processes.^[^ ^88^ ^]^ Under the condition of high alkalinity, TEOS undergoes a condensation reaction, leading to the formation of gripper‐like silicon species. These species then condense with Ti‐OH to form isolated Si‐O‐Ti. The matching hydrolysis rates of Si and Ti species contribute to the uniform distribution of Ti atoms.^[^ ^89^ ^]^

**Figure 10:**
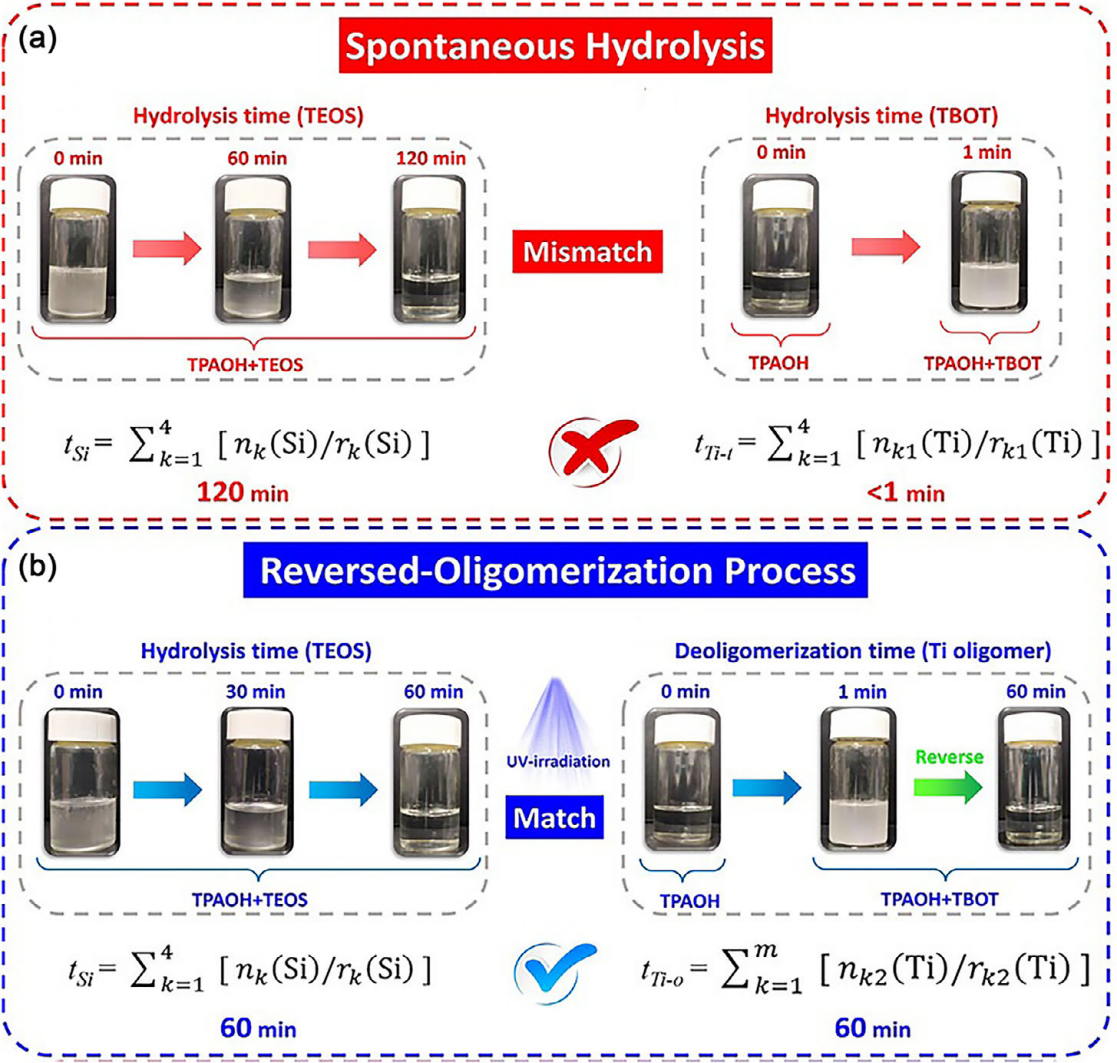
Hydrolysis of Ti and Si species in a) spontaneous and b) reversed‐oligomerization process. Reproduced with Permission.^[^ ^90^ ^]^ Copyright 2021, John Wiley & Sons, Inc.

(2) UV irradiation‐assisted approach: Lin et al.^[^ ^90^ ^]^ introduce the reversed‐oligomerization synthetic strategy to address the challenge of harmonizing the hydrolysis rates of Ti and Si precursors. UV irradiation can accelerate the dissociation of tetraethyl orthosilicate (TEOS) to Si monomers (Si(OH)_4_) (**Figure** **10**), while simultaneously slowing the deoligomerization of Ti oligomers, thus inhibiting the formation of anatase. When precursors were subjected to 60 min of UV radiation, TS‐1 without rutile was finally synthesized. This strategy facilitated by in situ generation of hydroxyl free radicals from UV irradiation is efficient, simple and eco‐friendly. It offers a novel, efficient and green synthesis idea for the industrial production of TS‐1molecular sieve.

(3) Prehydrolysis of silicate source: Geng et al.^[^ ^91^ ^]^ investigated the impact of TEOS prehydrolysis time on the distribution of Ti species in TS‐1 zeolites. Based on the characterization results from UV–vis and FT‐IR analyses, it was found that moderate prehydrolysis of TEOS effectively suppresses the formation of extra‐framework Ti and markedly boosts the framework Ti content. Specifically, the tetra‐coordinated framework Ti content reach nearly 100% when the hydrolysis of TEOS are carried out for 2.0–3.0 h. And that decrease to 93.5% and 96.9%, respectively, when shortening or extending the hydrolysis time of TEOS to 1 or 4 h. Notably, the TS‐1 sample with 2 h of prehydrolysis of TEOS exhibit more framework Ti and higher 1‐hexene conversion rate of 37.5% and and 1,2‐epoxy hexane selectivity of 85% than other TS‐1 with different TEOS prehydrolysis time. However, both excessively long and short prehydrolysis time are not conducive to hinder Ti incorporation into the framework, leading to a higher proportion of extra‐framework Ti species.

The hydrolysis rate matching strategy aims to synchronize the hydrolysis rate of Si and Ti source, thereby suppressing the generation of non‐framework Ti and facilitating an increase in the proportion of framework Ti. This approach facilitates the synthesis of TS‐1 molecular sieves with superior catalytic performance while also reducing production costs and paving the way for their industrial application. Nevertheless, achieving TS‐1 with a substantial presence of framework Ti species and high‐coordinated Ti species through this strategy remains challenging. Consequently, it is imperative to explore additional synthetic approaches to develop highly active TS‐1 catalysts, thereby advancing their industrial applications to a new level.

### 3.2 Crystallization Rate Matching Strategy

Besides the mismatch in hydrolysis rate of precursor, the mismatch in the doping rates of Si and Ti contributes to the emergence of non‐framework Ti species during the synthesis process. If crystallization proceeds too quickly, Ti^4+^ lacks sufficient time to be introduced into the framework. However, an overly sluggish crystallization rate may lead to the formation of anatase. By moderating the crystallization rate of TS‐1 (i.e., slowing down the crystallization rate of MFI molecular sieve), the incorporation rate of Ti into the framework can be effectively regulated. Ensuring that the rate of Ti entering the framework aligns with the rates of nucleation and crystallization can curb the formation of non‐framework titanium and boost the content of framework Ti species.

#### 3.2.1 Combined Solid–Liquid Phase Crystallization Method Promotes Formation of Rich Framework Titanium

In the liquid‐phase transformation mechanism, small crystal nuclei typically form first, followed by crystal growth within the liquid gel. Most Ti^4+^ is inserted into the lattice as crystallization nears completion.The solid‐phase transformation mechanism entails the direct crystallization of the amorphous solidified gel into titanium‐enriched TS‐1, thereby promoting the effective incorporation of titanium into the TS‐1 framework.^[^ ^92^ ^]^ The solid–liquid coupling mechanism describes the coexistence of solid and liquid phases during zeolite crystallization.

**Figure 11:**
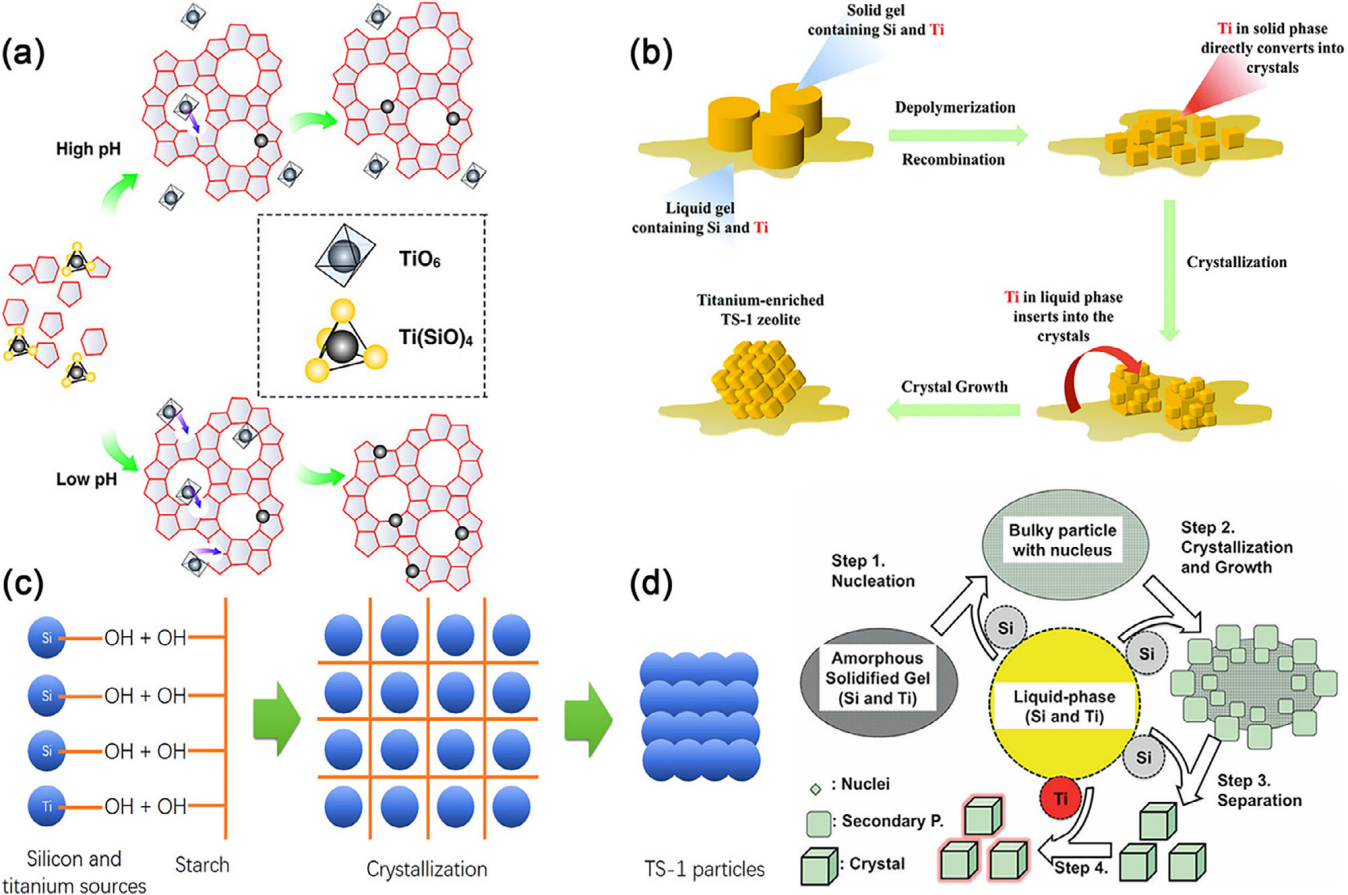
a)Possible formation combined mechanism of liquid‐phase and solid‐phase transformation in the formation of high‐performance TS‐1.Reproduced with permission.^[^ ^95^ ^]^ Copyright 2013, American Chemical Society. b) Scheme of the possible formation mechanism of enhanced‐titanium TS‐1. Reproduced with permission.^[^ ^96^ ^]^ Copyright 2021, Royal Society of Chemistry. c) TS‐1 with little extra framework Ti was hydrothermally synthesized using starch as the additive. Reproduced with permission.^[^ ^93^ ^]^ Copyright 2016, American Chemical Society. d) Possible formation combined mechanism of liquid‐phase and solid‐phase transformation in the formation of high‐performance TS‐1. Reproduced with permission.^[^ ^97^ ^]^ Copyright 2014, John Wiley & Sons, Inc.

Ammonium salts,^[^ ^85^, ^92^ ^]^ employed as crystallization modifiers, are utilized to lower the system's pH or solidify the gel. This ensures that the rate of Ti incorporation into the framework matches with the nucleation and crystallization rates, thereby inhibiting the formation of anatase TiO_2_ in TS‐1. An optimal amount of ammonium salt not only facilitates the regulation of the solid‐phase conversion mechanism, enabling more Ti to be incorporated into the framework, but also leads to the formation of Ti‐rich TS‐1 by suppressing rapid crystal growth. Consequently, Ti in liquid phase combine with more than four [SiO_4_] units, forming penta‐coordinated Ti. The addition of ammonium salts promotes the formation of a greater quantity of framework tetra‐coordinated Ti and a modest quantity of penta‐coordinated Ti, both of which function as active catalysts in selective oxidation reactions. Starch^[^ ^93^ ^]^ and sucrose^[^ ^94^ ^]^ release H^+^ through decomposition and carbonization during the crystallization process, lowering the pH value and enabling a good synchronization between the insertion rates of Ti and Si into the framework. Moreover, the addition of starch modifies the coordination state of titanium ions, slows down the crystallization rate of TS‐1, and ensures that the insertion rate of Ti aligns seamlessly with that of Si into the framework. An appropriate amount of starch can form a network structure in the aqueous solution. This structure effectively segregates the silicon and titanium sources into smaller fractions within the synthesis gel, impeding their combination, as depicted in **Figure** **11**c. TS‐1 with a starch/SiO_2_ mass ratio of 0.4 demonstrates a high value of 2.06 at I_960_/I_800_. In contrast, TS‐1 without any starch (starch content = 0) only exhibits a value of 1.56 at I_960_/I_800_, suggesting that starch addition enhances the Ti content within the framework and effectively eliminates non‐framework Ti. Moreover, during the synthesis process of TS‐1, the formation of starch micelles induces the creation of some mesopores. The mesoporous structure and abundant framework titanium significantly boost the catalytic activity of starch‐assisted TS‐1 in 1‐butene epoxidation. The starch‐assisted TS‐1 with a starch/SiO_2_ mass ratio of 0.4 achieves a TOF value of 637.9 mol H_2_O_2_ mol Ti^−1^ h^−1^ in 1‐butene epoxidation. reaction, whereas TS‐1 without starch only reaches 401.4 mol H_2_O_2_ mol Ti^−1^ h^−1^.

Glycine,^[^ ^95^ ^]^ 1,3,5‐benzenetricarboxylic acid (H_3_BTC)^[^ ^96^ ^]^ and polyacrylic acid (PAA)^[^ ^97^ ^]^ serve as additives to transform the precursor sol–gel into a solid–liquid mixed state, thereby regulating the crystallization process of TS‐1. This transformation reduces the crystallization rate and enhances the stability of the Si─O─Ti bond, effectively inhibiting the loss of tetrahedral coordination Ti species, inhibiting the formation of TiO_2_. (NH_4_)_2_CO_3_, glycine and H_3_BTC lower the pH value of the system and solidify the partially gel precursor (Figure 11a).^[^ ^95^ ^]^ The solid–liquid mixed‐phase transformation process yields a high content of framework Ti and a low content of extra‐framework Ti (Figure 11b).^[^ ^96^ ^]^ The Si/Ti ratio of TS‐1 via the solid–liquid mixed‐phase transformation process with the addition of H3BTC can reach as high as 48.5, whereas the Si/Ti ratio of traditional TS‐1 stands at only 79.0. In the 1‐hexene epoxidation reaction, TS‐1 synthesized via the solid–liquid mixed‐phase transformation process demonstrates a 1‐hexene conversion rate of 18.11%. This rate surpasses that of traditional TS‐1 (10.10%). The anionic polyelectrolyte PAA (Figure 11d)^[^ ^97^ ^]^ acting as gelating agent, partially converts the liquid‐phase precursor to the solid‐phase one. Framework tetra‐coordinated Ti is formed through direct conversion of Ti species from the amorphous solid phase precursor and the rapid insertion of Ti species from the liquid‐phase precursor into the lattice.

By appropriately decreasing the alkalinity of the synthesis sol–gel, the crystallization mechanism of TS‐1 can be shift to a solid–liquid mixed phase. This strategic adjustment not only effectively facilitates Ti incorporation into the framework, achieving a more even distribution of Ti within the framework and thereby enhancing the fundamental performance of the catalyst. Nevertheless, this approach also brings about issues of sluggish crystallization rates and extended preparation periods, which undoubtedly pose significant obstacles to the large – scale industrial production of the catalyst. At present, our understanding of the solid–liquid mixed phase transition mechanism remains rather limited. To address this, we can leverage advanced experimental design methods, such as the response surface methodology. Through this approach, an in‐depth analysis of the transition mechanism, as well as the intrinsic relationships between additive amounts, crystallization rates, and catalyst performance. Discover the optimal balance between crystallization rates and catalyst performance, ultimately provide a solid and reliable technical foundation for the industrial production of the catalyst.

#### 3.2.2 Zeolite Growth Modifiers Promotes Formation of Rich Framework Titanium

**Figure 12:**
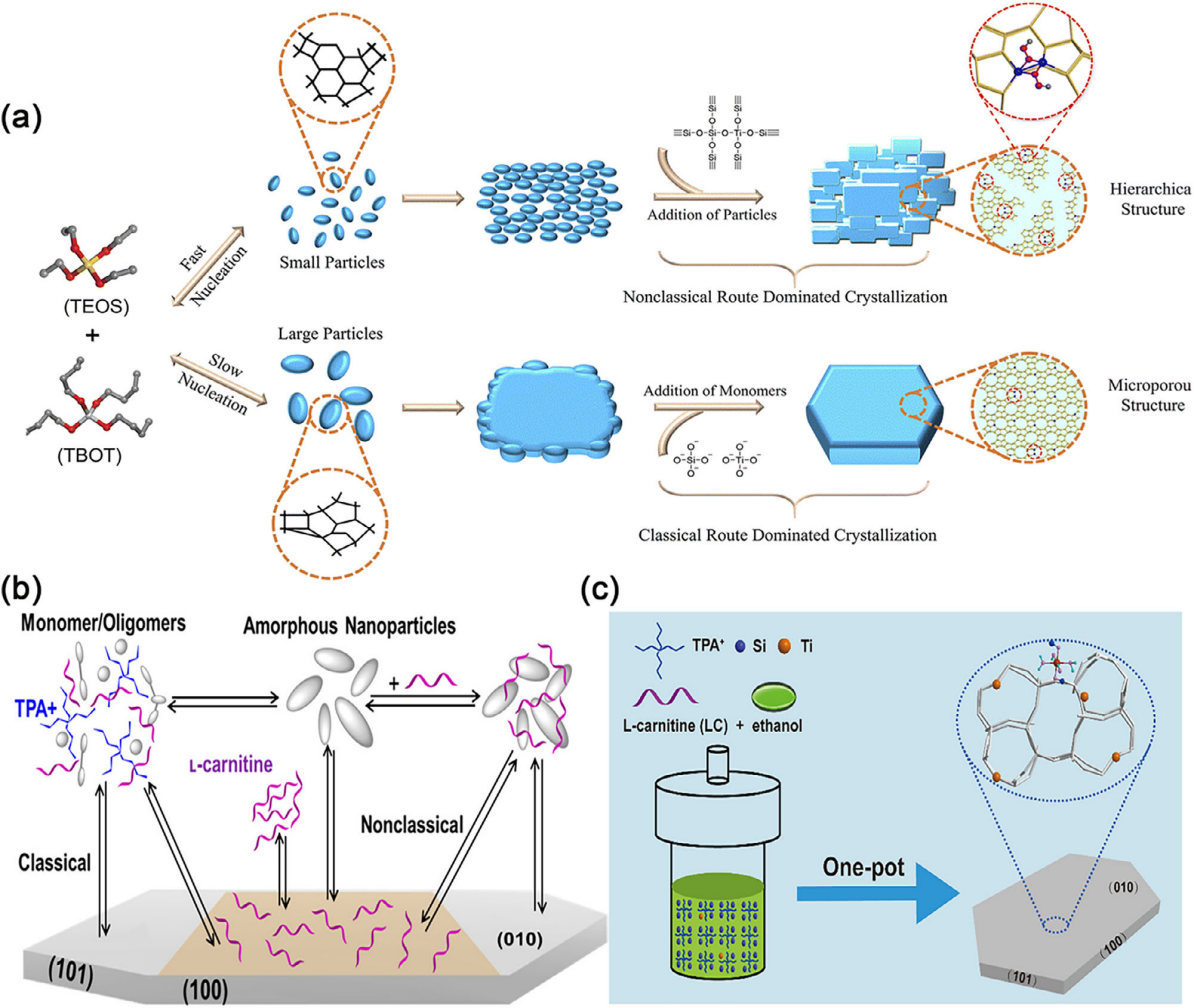
a) Proposed Schematic of the TS‐1 synthesis through classical or non‐classical crystallization pathways.Reproduced with permission.^[^ ^98^ ^]^ Copyright 2022, The Royal Society of Chemistry. b,c) Schematic of platelet TS‐1 in classical and non‐classical crystallization Pathways, mediated by L‐Carnitine. Reproduced with permission.^[^ ^99^ ^]^ Copyright 2020, American Chemical Society.

Zeolite synthesis generally proceeds via classical or non‐classical crystallization pathways.^[^ ^98^ ^]^ The classical route typically involves spontaneous nucleation, where monomers from the growth solution are added to the crystal surface through twisting, steps, and step positions, resulting in zeolite crystals with smooth surfaces. In contrast, non‐classical processes involve the addition and attachment of precursors, where these precursors can vary from oligomers to primary particles or even fully developed nanoparticles. These processes encompass disorder‐to‐order transformations, ultimately yielding crystals with irregular morphologies, rough surfaces, and mesoscopic structures (**Figure** **12**a).

Song et al.^[^ ^99^, ^100^ ^]^ demonstrates the formation process of TS‐1 via classical and non‐classical pathways. Initially, Si and Ti monomers or oligomers in the precursor gel aggregate to form amorphous nanoparticles. Subsequently, TS‐1 crystals gradually develop under the guidance of the TPAOH template. In the classical process, TS‐1 crystals exhibit a regular hexagonal prismatic shape, with an average size of 0.7 µm and a consistent thickness of 0.5 µm. In the non‐classical process, TS‐1 crystals grow through the accretion of small amorphous nanoparticles onto crystal faces. Here, L‐carnitine^[^ ^99^ ^]^ is introduced to form coordination bonds with monomer Si and Ti as well as amorphous nanoparticles, and it may function as an adsorbate covering the preferred ac crystal face. TS‐1 crystals formed via non‐classical pathways adopt a hexagonal plate‐like morphology (Figure 12b,c). The size of slender TS‐1 crystals are ≈0.8‐0.9 µm, and their growth along the b‐orientation significantly suppressed. In addition to framework TiO_4_, TS‐1 crystals synthesized by introducing L‐carnitine through a non‐classical pathways also contain abundant TiO_6_ species. When employed as a catalyst in 1‐hexene epoxidation, they can achieve a 55% conversion of 1‐hexene within 2 h, coupled with a 96% epoxy selectivity. In contrast, traditional TS‐1 exhibits a low 1‐hexene conversion rate of only 30% under the same conditions. Notably, the TOF of TS‐1 catalyst with a large amount of TiO_4_ and TiO_6_ can reach 131 h^−1^, double that of conventional TS‐1 (65 h^−1^). During non‐classical hydrothermal crystallization, the introduction of L‐carnitine not only modulates the anisotropic growth rate of TS‐1, but also promotes the uniform distribution of framework Ti by establishing suitable chemical interactions with Ti precursors. L‐Carnitine functions as a zeolite growth modifier in non‐classical hydrothermal crystallization processes. The function of zeolite growth modifiers is to inhibit crystal growth or promote crystal growth, dynamically regulate the growth rate of different crystal planes through specifically binding, thereby controlling the anisotropic growth of crystals. Yu et al.^[^ ^100^ ^]^ proposed strategies involving organic acids (L‐lysine) and seeds for synthesizing TS‐1 zeolites containing TiO_6_ (Ti(OSi)_2_(OH)_2_(H_2_O)_2_) and framework TiO_4_ (Ti(OSi)_4_) species. This synthesis method resulted in a 1‐hexene conversion rate of 33%, a higher turnover number (TON) value of 153, and comparable epoxy selectivity at 95%. In contrast, TS‐1 catalyst containing only TiO_4_ species, exhibited a 1‐hexene conversion rate of ≈22.5%. This synthetic approach offers a means to customize the quantity and distribution of titanium species within silica zeolites, thereby achieving superior catalytic performance across diverse processes.

Some polymers^[^ ^101^, ^102^, ^103^, ^104^ ^]^ are recognized as zeolite growth modifiers in directing non‐classical crystallization pathways, as their appropriate addition can selectively modify zeolite crystal morphology and size during synthesis.

**Figure 13:**
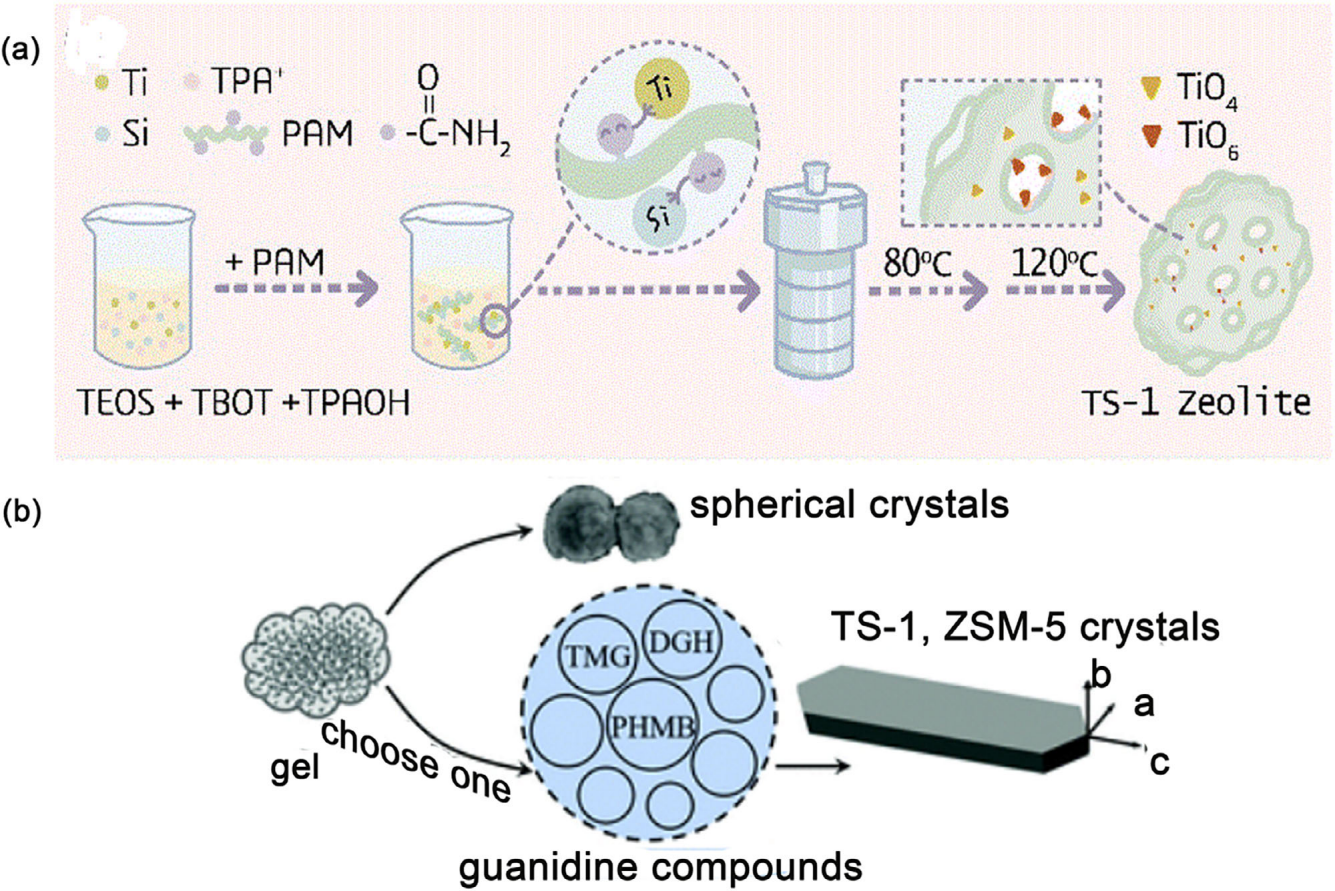
a) Schematic diagram of PAM‐assisted synthesis of TS‐1. Reproduced with permission.^[^ ^102^ ^]^ Copyright 2022, The Royal Society of Chemistry. b) Scheme of putative crystallization pathway of TS‐1 in the presence of guanidine compounds. Reproduced with permission.^[^ ^106^ ^]^ Copyright 2022, The Royal Society of Chemistry.

Zhang et al.^[^ ^102^ ^]^ utilized a polymer polyacrylamide (PAM) as zeolite growth modifiers to modulate the structural development of the TS‐1 zeolite precursor. The introduction of PAM accelerates nucleation and growing in non‐classical crystallization path and further refining the microenvironment around the active sites within the TS‐1 zeolite (**Figure** **13**a).

The acyl groups present on PAM engage in interactions with the hydrolysis products of TEOS, thereby promoting the spontaneous self‐assembly and precipitation of amorphous nanoparticles. These nanoparticles play a crucial role in facilitating the formation of zeolite crystal nuclei. The precise interaction between PAM and Ti enables the successful incorporation of Ti into TS‐1 in the form of TiO_6_. Additionally, the acyl groups on PAM establish hydrogen bonds with Si and Ti monomers as well as oligomers. This interaction aids in reaching facilitate the achievement of the critical aggregation concentration within the network region formed by the entanglement PAM long‐chain. This promotes the aggregation and participation of Si and Ti precipitates into worm like particles. The worm like particles drive the assembly of colloidal precursors with ordered units and stabilizing Ti species. Consequently, the crystallization time is halved, and the Ti content is enriched in TS‐1 (Si/Ti = 29). TS‐1 zeolite with high active mononuclear hexa‐coordinated Ti (TiO_6_) is prepared under the action of PAM, demonstrating improved catalytic efficacy in ODS of DBT and epoxidation of 1‐hexene. The results reveal that DBT conversion progressively increased with rising Ti content, particularly that of TiO_6_. When both TiO_4_ and TiO_6_ are present in TS‐1, it achieved complete removal (100.0%) of DBT molecules within 40 min, significantly outperforming that of traditional TS‐1 catalyst (≈10% of DBT).

A small amount of PEG additive^[^ ^103^ ^]^ can serve as a zeolite growth modifier to promote the swift nucleation and subsequent growth of the zeolites. The terminal hydroxyl groups of PEG may engage in interactions with the Si and Ti precursors during the processes of gelation and crystallization, thereby modulating the rate of Ti incorporation to be commensurate with that of Si. This leads to the to the formation of TS‐1 zeolites enriched framework Ti species and inhibiting the generation of anatase TiO_2_. The synthesized TS‐1 zeolites exhibit a notably higher 1‐hexene conversion rate of 52.0% and achieve 97.2% selectivity for ethylene oxide after a 2 h reaction. In comparison, the conventional TS‐1 only attains a 1‐hexene conversion rate of 34.0%. Under the test conditions, its TOF value reached 161 h^−1^, ≈1.7 times that of TS‐1 (TOF 9 h^−1^). Compared to conventional TS‐1, the PEG‐mediated TS‐1 exhibite superior catalytic activity in the epoxidation of various alkenes, including propylene, opentene, cyclohexene, and 1‐heptene.

Gelatin^[^ ^105^ ^]^ was added as zeolite growth modifiers to optimize TS‐1 morphology and the distribution of Ti. During the hydrothermal process, gelatin hydrolyzes into amino acids and carboxyl groups. The carboxyl groups lower the synthesis gel's pH, slowing nucleation rate. The amino groups are preferentially adsorb on the most active surface (0 10) of the MFI topology, thereby inhibiting TS‐1 growth along the b‐axis and forming b‐axis‐oriented TS‐1 plates. The TS‐1 with a n(Si/Ti) = 41.7, synthesized without gelatin addition, exhibits a prominent absorption peak at ≈320 nm in its UV–vis spectrum, confirming the presence of a substantial amount of anatase TiO_2_. In contrast, when TS‐1 with n(Si/Ti) = 46.4 was synthesized with an appropriate amount of gelatin, the absorption band at 320 nm, characteristic of anatase TiO_2_, vanishes. This indicates that the amino and carboxyl groups in gelatin can modulate the coordination state of Ti and suppress the formation of extra‐framework Ti species (octahedrally coordinated Ti and anatase TiO_2_).

The 2D growth of TS‐1 crystals^[^ ^106^ ^]^ are achieved by introducing guanidine compounds, (e.g., tetramethylguanidine, dodecylguanidine hydrochloride, as well as polyhexamethylene biguanidine hydrochloride) (Figure 13b). The amino or hydroxyl groups of these guanidine compounds preferentially adsorb onto the a‐c plane of MFI crystals, hindering growth along b‐axis. Consequently, continuous and dense b‐axis‐oriented molecular sieves are formed, effectively shorting the molecular diffusion pathway. Tetramethylguanidine additionally decelerate slows crystallization and modulate the rate of titanium incorporation into the framework, allowing more titanium to enter the MFI framework and thereby increasing the framework Ti content in the molecular sieve.

**Figure 14:**
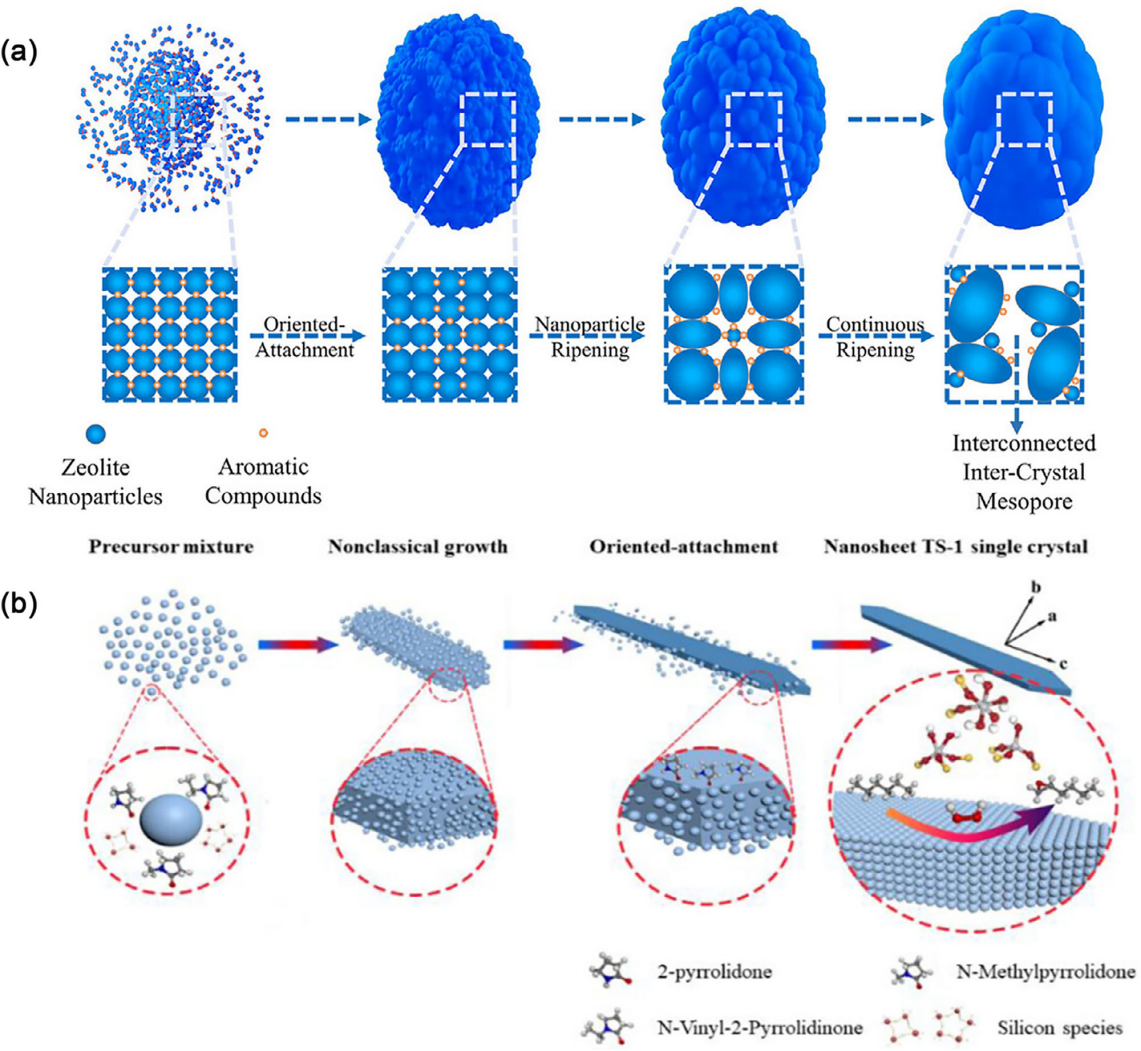
Evolution Process of TS‐1 crystals with a) aromatic compounds. Reproduced with permission.^[^ ^107^ ^]^ Copyright 2023, John Wiley & Sons, Inc. b) Evolution Process of TS‐1 crystals with pyrrolidone compounds. Reproduced with permission.^[^ ^108^ ^]^ Copyright 2025, American Chemical Society.

Aromatic compounds^[^ ^107^ ^]^ including p‐Phthalic acid, 2,5‐Dihydroxyterephthalic acid, and 2,5‐Diaminoterephthalic acid can serve as zeolite growth modifiers to facilitate rapid nucleation and growth. Their terminal hydroxyl groups interact with Si and Ti precursors, thereby increasing tetra‐coordinated framework Ti content and suppressing anatase TiO_2_ formation (**Figure** **14**a). Machine‐learning strategy was employed to investigate and analyze the key factors inhibiting anatase TiO_2_ in hierarchical TS‐1. Eight models were utilized to examine the effects of 12 features, achieving over 80% accuracy in predicting anatase formation. Pyrrolidone compounds^[^ ^108^ ^]^ (e.g., 2‐pyrrolidone, N‐methylpyrrolidone, and N‐vinyl‐2‐pyrolidinone) are effective in regulating TS‐1 nanosheet synthesis as zeolite growth modifiers (Figure 14b). Short b‐axis TS‐1 nanosheets containing abundant TiO_6_ species are synthesized through non‐classical process. These compounds adsorb onto the (010) crystal surface, controlling anisotropic growth. The abundant TiO_6_ species and high diffusion rate endow short b‐axis TS‐1 with outstanding catalytic performance in olefin epoxidation reactions. These nanoparticles demonstrate a 1‐hexene conversion rate of 39.62% and a 1,2‐epoxyhexane selectivity of 92.1%. Nevertheless, the selectivity of TS‐1 nanoparticles synthesized via this strategy is marginally lower than that of conventional TS‐1. This discrepancy primarily arises from the presence of numerous high‐coordination titanium active species in the TS‐1 catalyst synthesized using pyrrolidone compounds. These active species proceed to catalyze the oxidation of epoxy hexane, thereby causing a slight reduction in selectivity.

The interaction of molecular modifier with Si and Ti species facilitates colloidal precursor assembly, and enhances the stability of Ti species within it. This pathway shortens crystallization time, produces abundant TiO_6_/TiO_4_ species, and enhances the catalytic performance in selective oxidative reaction. Compared with the traditional synthesis pathway, controlling TS‐1 zeolites morphology and Ti coordination states by adding crystal growth modifier is more efficient and simpler. This method holds promise for developing high‐efficient titanium silicate catalysts.

The crystallization of TS‐1 with zeolite growth modifiers exhibits non‐classical mechanism characteristics, leading to an effective enhancement in both the physical and chemical attributes of the zeolite crystals. Zeolite growth modifiers (such as amines, polymers, etc.) can bind to specific crystal faces or react with amorphous precursors, selectively altering the morphology and size, as well as the growth rate of crystals and Ti incorporation to align with the doping rate of Si. This results in the formation of TS‐1 zeolite rich in tetra‐ coordinated framework Ti. The introduction of these modifiers can effectively regulate the morphology and active titanium species of TS – 1 zeolites, opening up new avenues for the development of highly efficient titanium – silicon zeolites. However, these modifiers often lead to the formation of worm‐like or plate shaped TS‐1, their relatively large size results in long internal diffusion paths for reactants and products, resulting a detrimental impact on catalytic performance. Therefore, it is of utmost importance to precisely control the crystallization process, regulate the size and dimension of zeolites, and synthesize 2D TS‐1 with a ultathin nanosheets. These regulation of these 2D nanosheets ensures a high degree of uniformity and accessibility in the distribution of active titanium species.

#### 3.2.3 Controlling Crystallization Kinetics

Zeolite crystallization comprises two primary stages: nucleation and crystal growth, both of which significantly directly influence the structure and catalytic performance of zeolite. An excessively slow crystallization rate leads to low crystalline, whereas an overly rapid rate causes a significant mismatch between MFI structure formation rate and Ti^4+^ insertion rate, resulting in more TiO_2_.

**Figure 15:**
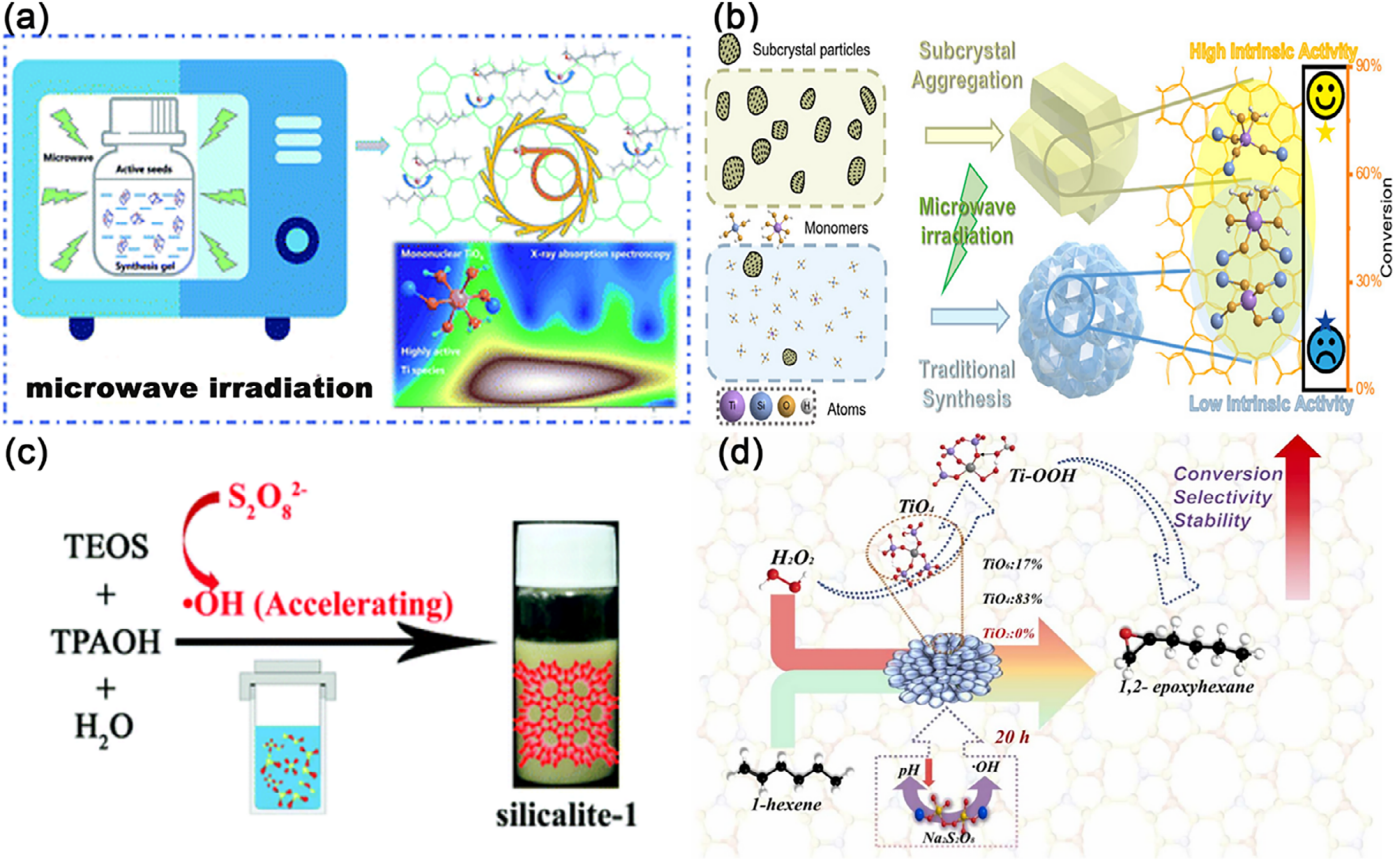
a) Synthesis of zeolite molecular sieves by a seed‐assisted and microwave irradiation route permission. Reproduced with permission.^[^ ^109^ ^]^ Copyright 2002, The Royal Society of Chemistry. b) Synthesis of TS‐1 with active hydrogen‐bonded Ti species through subcrystal aggregation crystallization and microwave irradiation route. Reproduced with permission.^[^ ^111^ ^]^ Copyright 2023, American Chemical Society. c) Synthesis process of TS‐1 by hydroxyl radical route. Reproduced with permission.^[^ ^113^ ^]^ Copyright 2018, The Royal Society of Chemistry. d) Synthesis of TS‐1 anatase free by hydroxyl radical route. Reproduced with permission.^[^ ^114^ ^]^ Copyright 2024, Elsevier B.V.

The microwave‐assisted hydrothermal synthesis route^[^ ^109^, ^110^ ^]^ can expedite the crystallization rate, reduce energy consumption, ensure uniform nucleation and facilitate rapid production. Ti‐enriched TS‐1 can be achieved within 30 to 90 min through microwave irradiation (**Figure** **15**a).^[^ ^109^ ^]^ TS‐1 containing mononuclear hexa‐coordinated TiO_6_ species^[^ ^110^ ^]^ can be rapidly synthesis via active seed‐assisted microwave radiation route. Under microwave irradiation, more highly coordinated Ti species provided by active crystal seeds are strongly activated, forming stable mononuclear TiO_6_. In the epoxidation of 1‐hexene, TS‐1 containing abundant TiO_6_ species through an active seed‐assisted microwave irradiation route exhibits high catalytic activity. The conversion of 1‐hexene on TS‐1 reaches 28.0%, markedly higher than that of conventionally TS‐1 (18.8%). Moreover, the TiO_6_ species endow TS‐1 with a 1‐hexene TON of up to 272, nearly 70% greater than that of conventional TS‐1 (161). Upon further aggregation and crystallization of subcrystals via microwave irradiation, highly coordinated TiO_x_ (x > 5) and penta‐coordinated TiO_5_ (Ti(OSi)_3_(OH)(H_2_O)) with one Ti‐OH groups emerge in TS‐1 (Figure 15b).^[^ ^111^ ^]^ The TS‐1 catalyst containing active TiO_5_ with Ti‐OH exhibits superior catalytic activity in light olefin epoxidation compared to traditional TS‐1. The inherent activity of Ti‐OH endows TS‐1 with exceptional catalytic performance, enabling it to achieve an impressive 72% conversion rate of allyl chloride during epoxidation. This performance markedly surpasses the 19% and 32% conversion rates demonstrated by TS‐1 zeolites containing TiO_4_ and TiO_6_ active species, respectively. Moreover, TS‐1 comprising TiO_5_ and Ti‐OH achieves a turnover number (TON) for allyl chloride of up to 340, a figure nearly 70% higher than that of TS‐1 containing TiO_4_ and TiO_6_.

Hydroxyl radicals assisted seed hydrolysis route^[^ ^112^, ^113^, ^114^ ^]^ can expedite crystallization and accomplish the higher yields of zeolites. The ·OH radical can remarkably accelerate the reconstruction of Si─O─Si bond and polymerization of Si species during zeolite nucleation. Sulfate radicals (SO_4_·^2−^), generated from sodium persulfate (SPS)^[^ ^113^, ^114^ ^]^ via S_2_O_8_^2−^, rapidly react with OH^−^ to produce ·OH radicals. The ·OH radical can accelerate the crystallization of TS‐1. The addition of SPS lowers the pH value of the precursor, avoiding the excessive crystallization (Figure 15c). This reduction in pH is conducive to promoting Ti incorporation into framework and inhibiting the formation of anatase TiO_2_. Under the assistance of SPS, a fraction of titanium more active TiO_6_ species (Figure 15d). TS‐1 prepared with the aid of SPS demonstrates a Si/Ti ratio as low as 31 and achieves a 20% conversion rate in the epoxidation of 1‐hexene. This conversion rate represents a 200% increase compared to TS‐1 synthesized without the addition of SPS. Furthermore, employing a hydroxyl radical‐assisted approach enables the efficient and rapid synthesis of TS‐1 devoid of anatase TiO_2_.

The dynamic regulation strategy for crystallization controls nucleation and growth kinetics, thereby influencing the growth rate and crystallization behavior of zeolite crystals, and ultimately modulating their morphology and Ti coordination state properties.^[^ ^115^ ^]^

**Figure 16:**
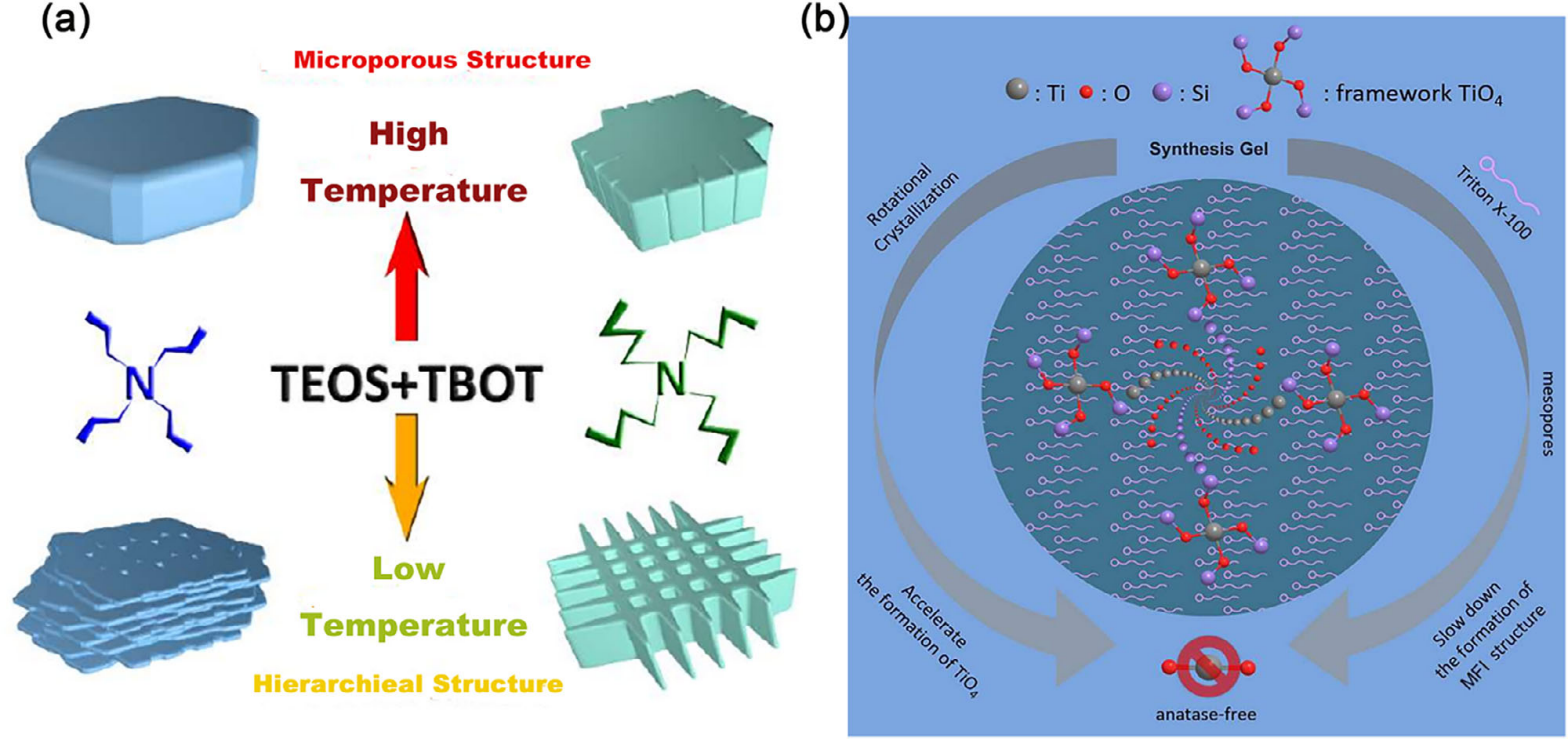
a) Synthesis of Anatase‐free hierarchical TS‐1 by two‐step hydrothermal crystallization strategy. Reproduced with permission.^[^ ^116^ ^]^ Copyright 2020, The Royal Society of Chemistry. b) Synthesis of anatase‐free hierarchical TS‐1 under rotational crystallization conditions.Reproduced with permission.^[^ ^118^ ^]^ Copyright 2018, The Royal Society of Chemistry.

Anatase‐free hierarchical TS‐1^[^ ^116^ ^]^ zeolite under dynamic crystallization via a two‐stage hydrothermal crystallization approach can be constructed (**Figure** **16**a). The two step crystallization involves two processes: nucleation at a low temperature, followed by crystallization and growth at a slightly elevated temperature. The low crystallization temperature employed in this approach effectively inhibits the formation of anatase TiO_2_, and limit the excessive growth of 2D layers, promoting layered growth of TS‐1, thereby facilitating the construction of abundant hierarchical pore structures. The stepwise crystallization approach not only facilitates the incorporation of a greater amount of Ti species into TS‐1 crystals, but also enhances the formation of hierarchical pore structures. Consequently, the oxidant efficiency is improved and the catalytic performance of the TS‐1 catalyst in the ODS of DBT are greatly enhanced via stepwise crystallization. Through stepwise crystallization, the hierarchical TS‐1 catalyst achieves a complete 100% conversion of dibenzothiophene (DBT) in a mere 10 min. In contrast, under the same reaction conditions, traditional microporous TS‐1 only manages a 12% DBT conversion. Additionally, the hierarchical TS‐1 exhibits an outstanding oxidant utilization efficiency of 80.8%, significantly outperforming the 21.7% efficiency demonstrated by the traditional microporous TS‐1. This enhanced performance is attributed to the abundant mesoporous structure and the absence of anatase, factors that collectively boost both its catalytic activity and oxidant utilization efficiency. The temperature‐controlled crystallization technique offers a fresh perspective for fabricating hierarchical zeolites free from anatase by precisely controlling the crystal growth process.^[^ ^117^ ^]^

Under rotational crystallization conditions,^[^ ^118^ ^]^ nano‐sized anatase‐free hierarchical TS‐1 is successfully synthesized using Triton X‐100 as a mesoporous template (Figure 16b). TS‐1 prepared by rotational crystallization method has a higher framework Ti content (I_960/800_ = 1.78) than that by the static crystallization method (I_960/800_ = 1.63). Rotational crystallization speeds up the integration process of Ti, aligning it closely with the incorporation rate of Si (I_960/800_ = 1.78), thereby effectively preventing the generation of anatase TiO_2_.

Kinetic control strategies such as microwave‐assisted hydrothermal synthesis, hydroxyl radical and seed‐assisted synthesis, multi‐step and stepwise hydrothermal synthesis, and rotational crystallization, etc., are used through effective control of nucleation and crystal growth, achieving the synthesis of TS‐1 with diverse titanium active species and anatase‐free, paving the way for new applications in molecular selective oxidation reactions. However, the industrial implementation of these synthetic strategies still needs further laboratory exploration.

#### 3.2.4 Continuous Flow Synthesis Route

**Figure 17:**
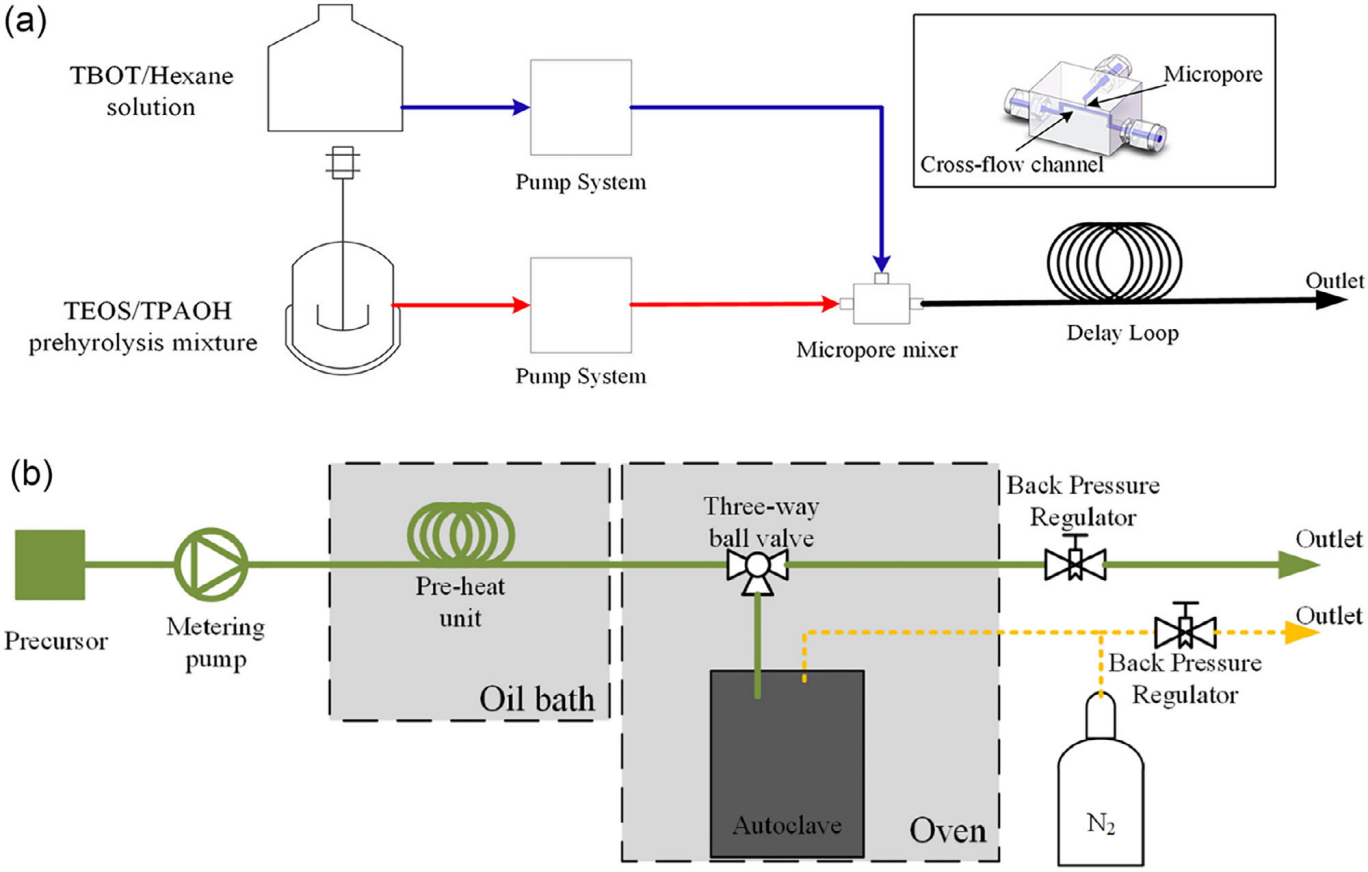
a) Schematic overview of micro‐sieve pore reactor system. Reproduced with permission.^[^ ^119^ ^]^ Copyright 2019, American Chemical Society. b) Schematic overview of flow system. Reproduced with permission.^[^ ^120^ ^]^ Copyright 2019, Elsevier B.V.

To expedite the synthesis process, researchers turned to specialized reaction apparatus like flow systems and microwave reactors for the preparation of TS‐1.^[^ ^119^, ^120^ ^]^ TS‐1 free of extra‐framework Ti species can be synthesized using liquid–liquid microdispersion method (**Figure** **17**a).^[^ ^119^ ^]^ In this approach, TBOT in n‐hexane droplets is uniformly distributed within a prehydrolyzed oligosilicate mixture inside a microporous reactor. Precise control of the feed rate not only swiftly shortens precursor crystallization time but also strengthens interactions among Ti, Si species, and templates, curbing TiO_2_ formation. The employed solvent, n‐hexane, can be easily recovered via phase separation, thus cutting down energy use. Through precise control of the precursor solution state through a liquid–liquid micro‐dispersion process, TS‐1 with a remarkably low Si/Ti molar ratio of 51.5 can be synthesized, and this variant is devoid of non‐framework Ti. This Si/Ti ratio shows a significant reduction when compared to the typical ratio of 54.6 found in traditional TS‐1. This continuous microdispersion technique offers a scalable, controllable and reliable amplification for the preparation of TS‐1. Hu et al.^[^ ^120^ ^]^ exploit a swift and continuous synthesis of TS‐1 nanoparticles by utilizing a flow system comprising a tubular reactor and an autoclave (Figure 17b).

The continuous flow method enables the rapid synthesis of TS‐1 nanoparticles with abundant framework Ti species within just a few min. This technology ensures the absence of non‐framework Ti species, as rapid nucleation of the precursor. Compared to traditional crystallization processes, the continuous flow process effectively avoids the decomposition of TPAOH and maintains alkali environment throughout the prolonged hydrothermal process. Therefore, the continuous flow method stands out for its highly efficiency, greener, and low energy consumption.

Strategic management of the quantity and coordination states of active Ti species within zeolites is pivotal for optimizing catalytic performance. A straightforward approach to augment the catalytic activity of TS‐1 involves elevating its Ti content within the framework. However, the disparity in crystallization rates between Si and Ti often culminates in the undesirable formation of anatase TiO_2_ species, subsequently diminishing the framework tera‐coordinated Ti content. To address this issue, certain measures are employed to regulate the crystallization process of TS‐1 zeolite. For instance, zeolite growth regulators are utilized, steering the transition of the crystallization mechanism from a liquid‐phase mechanism to a solid–liquid coupled mechanism, and to fine‐tune the crystallization kinetics. These strategic intervention achieve precise control over the crystallization pathway, facilitate the construction and regulation of the formation of Ti active species, effectively increase the content of tera‐coordinated framework Ti, and minimize the formation of anatase TiO_2_.

Part of TS‐1 zeolite synthesized via a crystallization regulation process exhibits a slender or planetary morphology, however, its thickness constrains the effective catalytic performance of the active Ti species situated within the crystal. Through precise regulation of the crystallization process, control over the zeolite dimensions, and synthesis of 2D TS‐1 nanosheets, a high degree of uniformity in the distribution of active Ti species can be achieved. This uniformity is advantageous for fully harnessing their catalytic potential in oxidation reactions.

### 3.3 Dissolution‐Recrystallization Strategy

The typical micropores in TS‐1 seriously hinder mass transfer of macromolecules during their oxidation reaction. High accessible Ti content in TS‐1 zeolite is key to enhancing selective oxidation catalytic performance. The dissolution‐recrystallization approach represents a promising strategy for synthesizing mesoporous zeolite, effectively overcoming the limitation of the mass transfer by micropores and improve the accessibility of active Ti species.

**Figure 18:**
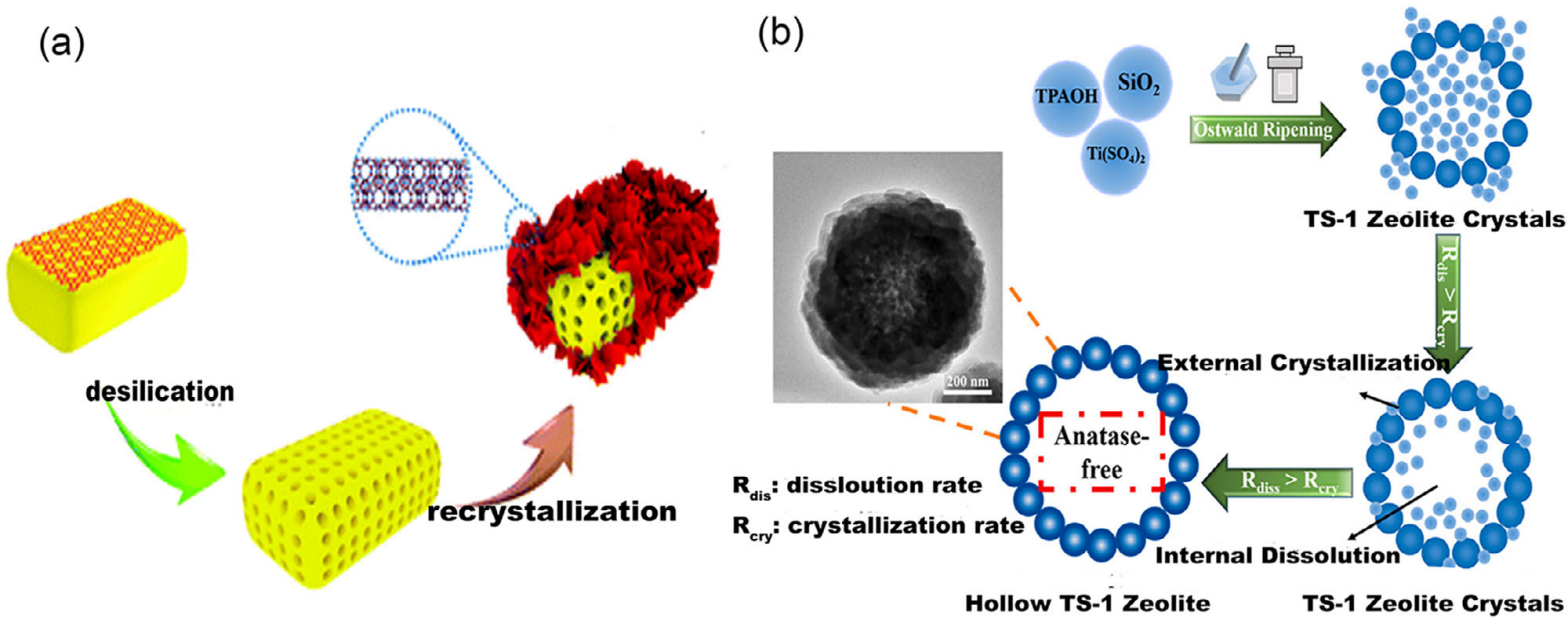
a) Mechanism of dissolution‐recrystallization in Alkaline Treatment of Zeolite. Reproduced with permission.^[^ ^121^ ^]^ Copyright 2019, American Chemical Society. b) Synthesis of Hollow TS‐1 by via dissolution‐recrystallization process. Reproduced with permission.^[^ ^123^ ^]^ Copyright 2022, Elsevier B.V.

The approach involves controlled desilication and recrystallization.^[^ ^121^, ^122^, ^123^, ^124^ ^]^ The formation of mesopores is closely related to the desilication process, the OH^−^ ions diffuse into the channels and erode the inner part of the crystals, causing the dissolution of Si and destruction of zeolite framework (**Figure** **18**a).^[^ ^121^ ^]^ The recrystallization of dissolved substances on the surface of zeolite enhances its crystalline (Figure 18b).^[^ ^123^ ^]^ Zhai et al.^[^ ^124^ ^]^ investigate the dissolution and absorption mechanism related to the formation of mesopores by DFT calculation. This mechanism arises from the dynamic competition between surface crystallization and internal dissolution rates throughout the dissolution‐recrystallization process. When the internal dissolution rate slightly outpaces that of surface crystallization, the hollow structure can be achieved, offering a new pathway for synthesizing hierarchical zeolite. This process also alters the distribution of titanium species in TS‐1. TPAOH is commonly employed in the dissolution‐recrystallization process process of zeolites.^[^ ^125^ ^]^ A low concentration TPAOH solution can reduce the content of tetra‐coordinated titanium due to its simultaneous dissolution with silicon and formation of anatase. Conversely, a relative high concentration of TPAOH facilitates the redistribution and reinsertion of anatase and extra‐framework Ti species into the framework via dissolution‐recrystallization, minimizing anatase formation.^[^ ^126^, ^127^ ^]^ TPA^+^ with a kinetic diameter of 0.86 nm, is unable to diffuse into the channel and instead deposits on the surface. This deposition effectively prevents the erosion of the zeolite shell. The high concentration of TPA^+^ accelerates surface crystallization, facilitating the recrystallization of extra‐framework Ti species into more tetra‐coordinated framework Ti.

**Figure 19:**
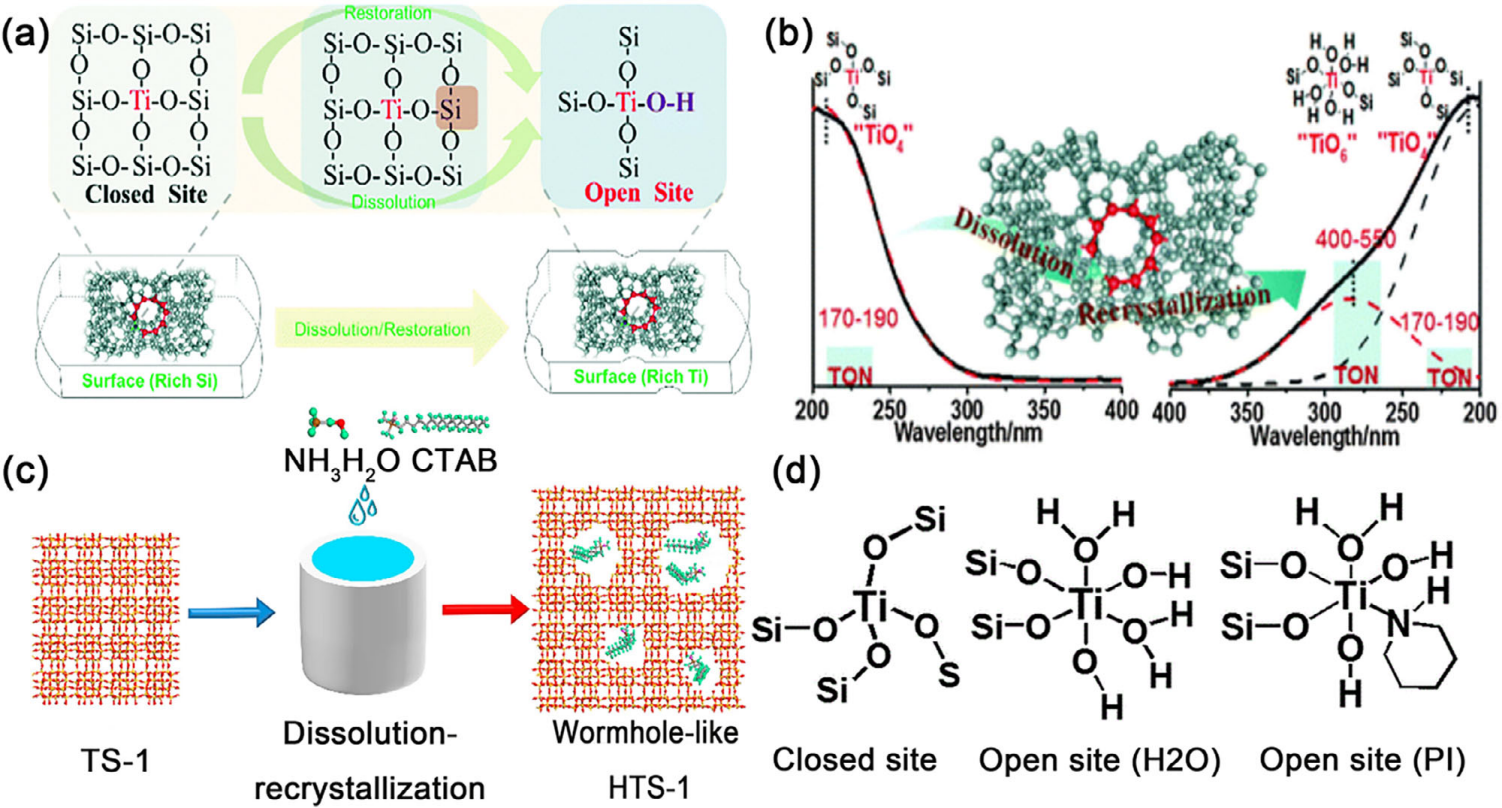
a) Scheme for constructing open defective Ti(OSi)_3_OH in TS‐1. Reproduced with permission.^[^ ^132^ ^]^ Copyright 2018, The Royal Society of Chemistry. b) Evolve of framework Ti(OSi)_4_ to mononuclear TiO_6_ species. Reproduced with permission^.[^ ^129^ ^]^ Copyright 2016, The Royal Society of Chemistry. c) Synthesis of the HTS‐1 zeolite. Reproduced with permission.^[^ ^130^ ^]^ Copyright 2018, Elsevier B.V. d) Evolve of framework Ti(OSi)_4_ to hexa‐coordinated Ti(OH)_2_(OSi)_2_(H_2_O)_2_ and Ti(OH)_2_(OSi)_2_(H_2_O)(PI) active species in TS‐1. Reproduced with permission.^[^ ^133^ ^]^ Copyright 2015, The Royal Society of Chemistry.

It has been discovered that the type and quantity of bases and templates employed in the dissolution‐recrystallization process not only influence the proportion of tetra‐coordinated Ti in TS‐1 but also plays a pivotal role in generating highly coordinated new Ti species. The catalytic performance of TS‐1 is improved upon treatment with TPAOH, primarily due to the formation of penta‐coordinated Ti.^[^ ^128^ ^]^ TPA^+^ selectively dissolves framework Si in alkaline media to regulate Ti's chemical environment and promote recrystallization (**Figure** **19**b).^[^ ^129^ ^]^ Furthermore, the integration of TPA^+^ with other additives enables fine‐tuning of the dissolution–recrystallization equilibrium within TS‐1, thus achieving a more precise control over pore architecture and Ti active centers.^[^ ^130^, ^131^, ^132^ ^]^

During chemical post‐treatment of TS‐1, dissolution and recrystallization may occur separately or concurrently either on the surface or within the TS‐1 crystals. The treatment may not only change the nature of Ti species but also redistribute them. A variety of bases and templates can be used to form defective tetra‐coordinated Ti(OSi)_3_OH in TS‐1 crystals via dissolution‐recrystallization. A high concentration of TPAOH^[^ ^128^ ^]^ and ethylamine^[^ ^132^ ^]^ facilitates the transformation of Ti(OSi)_4_ into Ti(OSi)_3_OH. This transformed species shows superior catalytic oxidation capabilities compared to other Ti species in epoxidation process of 1‐hexene. During dissolution, an O─Si bond within Ti(OSi)_4_ breaks and is replaced by an OH bond. Ethylamine can selectively dissolve the Si species around Ti(OSi)_4_ on the surface of TS‐1 crystals, leading to their subsequent conversion into the defective Ti(OSi)_3_OH (Figure 19a). In the propylene epoxidation reaction, the yield of propylene oxide significantly increases from 49.2% to 82.2%, owing to the formation of Ti(OSi)_3_OH on TS‐1. Meanwhile, the lifetime of TS‐1 containing defective Ti(OSi)_3_OH is prolonged from 20 h (conventional TS‐1) to 45 h in continuous cyclohexanone ammoximation. Wu et al.^[^ ^132^ ^]^ employ Gaussian fitting combined with UV/Vis spectroscopy to separately evaluate the individual catalytic contributions of Ti(OSi)_4_, Ti(OSi)_3_OH, and TiO_6_ species in 1‐hexene epoxidation. The TON values for Ti(OSi)_3_OH species can reach 864, while the TON values for Ti(OSi)_4_ and TiO_6_ species are 178 and 535, respectively. These findings indicate that, in the 1‐hexene epoxidation reaction, the catalytic performance order of Ti species is Ti(OSi)_3_OH>TiO_6_>Ti(OSi)_4_. By employing ethylamine (EA) and tetrapropylammonium bromide (TPABr)^[^ ^130^ ^]^ to regulate the balance between selective dissolution capability of Si species and recrystallization, highly active TiO_6_ species were obtained. TS‐1 containing TiO_6_ exhibits as high as 50% conversion of 1‐hexene compared to conventional TS‐1. In the epoxidation of 1‐hexene, the TON value of TiO_6_ species in TS‐1 is 2–3 times higher than that of TiO_4_ species. This indicates that the TiO_6_ species in TS‐1 has a superior catalytic oxidation activity than TiO_4_ in epoxidation of 1‐hexene.

TS‐1 molecular sieves (HTS‐1) with wormhole‐like structures of ≈45 nm were successfully synthesized via a dissolution‐recrystallization process using a combination of cetyltrimethylammonium bromide (CTAB) and ammonia solution (Figure 19c).^[^ ^132^ ^]^ During recrystallization, the Si‐OH species in HTS‐1 were transformed into Si‐O‐Ti species. This alteration not only enhanced the hydrophobicity of the support but also improved the stability of olefin epoxidation. Wu et al.^[^ ^133^ ^]^ successfully synthesized a novel hexa‐coordinated titanium species, Ti(OSi)_2_(OH)_2_(H_2_O)_2_, by introducing an organic amine ligand, piperidine (PI), into the post‐treatment solution. The ligand PI coordinated with Ti(OSi)_4_ forming Ti(OSi)_2_(OH)_2_(H_2_O)PI, then the organic ligand PI was replaced by water molecules, yielding a new hexa‐coordinated titanium species Ti(OSi)_2_(OH)_2_(H_2_O)_2_ after calcination (Figure 19d). The two hexa‐coordinated titanium species exhibited enhanced selectivity for propylene oxide (PO) and recyclability in the olefin epoxidation reaction. Replacing ligand PI with hexamethyleneimine (HMI) can form hexa‐coordinated titanium Ti(OSi)_2_(OH)_2_(H_2_O)HMI.The strategy of combining with an organic ligand via the dissolution‐recrystallization process for generating hexa‐coordinated titanium paves the way for controlling the coordination environment of titanium species through appropriate modifications.

The dissolution‐recrystallization process is conducted using TPAOH and ammonium salt or NH_3_·H_2_O, facilitating the formation of highly coordinated Ti species.^[^ ^134^, ^135^, ^136^, ^137^, ^138^ ^]^ Simultaneously, hierarchical TS‐1, featuring with surface grooves, intracrystal voids, and secondary pore, is thus formed. Ammonium salts and OH^−^ selectively dissolve the Si from the external and internal surfaces of TS‐1, respectively. In the composite system of TPAOH and (NH_4_)_2_CO_3_,^[^ ^136^ ^]^ TPAOH facilitates the conversion of Ti(OSi)_4_ into Ti(OSi)_3_(OH) and the formation of cavities. NH_3_ derived from (NH_4_)_2_CO_3_ serves as an electron donor, occupying the vacant orbitals of tetra‐coordinated Ti. Meanwhile, OH^−^ attacks the framework Si, promoting the formation of Ti─OH bonds. At this process, intermediate species Ti(OSi)_x_(OH)_4_‐_x_(NH_3_)_2_ are yielded, and they subsequently undergo a transformation into Ti(OSi)_2_(OH)_4_ during the final calcination step. The secondary pore and high‐coordinated Ti species are beneficial to the epoxidation of cyclohexene, achieving a cyclohexene conversion rate of 45.4% and an epoxycyclohexane selectivity of 94.5%.^[^ ^135^ ^]^ These performance metrics surpass those of untreated TS‐1 (19.0% and 80.4%).

Due to its relatively large size, TPA^+^ are confined to the exterior surface of TS‐1. These ions combines with titanium accompanied by desilication and detachment, leading to the formation of form highly coordinated titanium species during the recrystallization process. While cavities are formed within the internal pores, thereby improving the diffusion and accessibility of active Ti species. The synergistic effect between highly coordinated and tetra‐coordinated titanium species, along with the presence of numerous internal cavities, endows TS‐1 with excellent performance in reactions such as phenol hydroxylation, 1‐octene epoxidation and conversion of Ethylene to Glycol.

**Figure 20:**
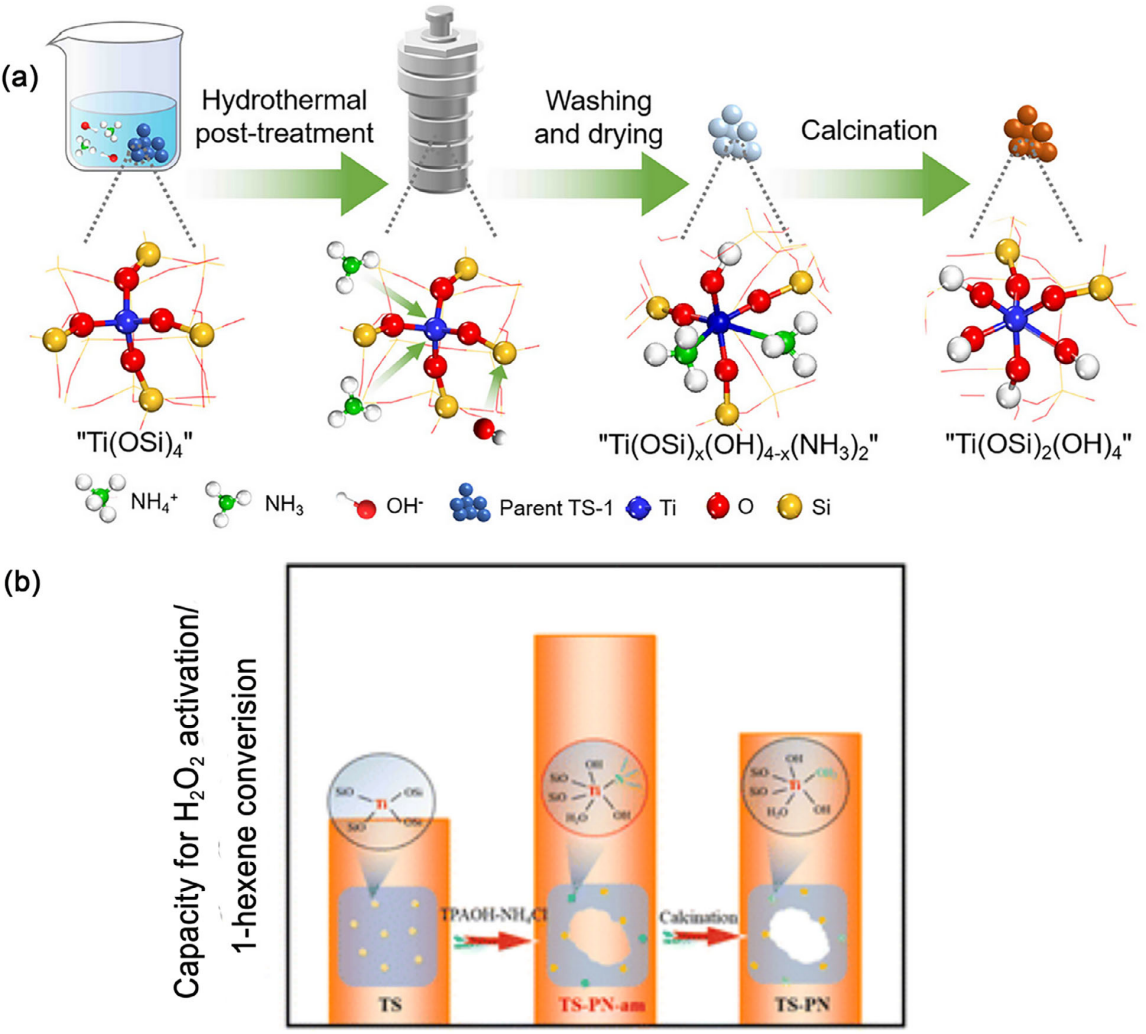
a) Formation mechanism of Ti‐VI during TPAOH and ammonium salt‐treatment of TS‐1. Reproduced with permission.^[^ ^137^ ^]^ Copyright 2024, Elsevier B.V. b) Catalytic performance of TS‐1 with Ti(OSi)_2_(OH)_2_(H_2_O)_2_. Reproduced with permission.^[^ ^138^ ^]^ Copyright 2025, John Wiley & Sons, Inc.

Beyond TPAOH, bases like EA, triethylamine, and NH_3_·H_3_O also enable dissolution and recrystallization of titanium silicate molecular sieves, yielding highly coordinated titanium species and secondary pores. It has been demonstrated that NH_3_·H_2_O can disrupt the charge balance constraint of TPA^+^, thereby aiding the implantation of TPAOH in pores during the recrystallization process. Furthermore, the presence of NH_3_·H_2_O accelerates both the dissolution and recrystallization process, though it concomitantly introduces extra‐framework titanium species. When the NH_3_·H_3_O are replaced by ammonium salt,^[^ ^136^, ^138^ ^]^ more penta‐coordinated or hexa‐coordinated Ti species that are favorable for epoxidation reactions, is observed. When TPAOH and NH_4_Cl are employed to treat TS‐1 via the dissolution‐recrystallization process, a novel hexa‐coordinated Ti species including an organic amine ligand, Ti(OSi)_2_(OH)_2_(H_2_O)TPA, can be formed (**Figure** **20**a).^[^ ^137^ ^]^ Upon calcination to eliminate the organic components, Ti(OSi)_2_(OH)_2_(H_2_O)TPA transforms into Ti(OSi)_2_(OH)_2_(H_2_O)_2_. The two hexa‐coordinate Ti species markedly enhances the activation of H_2_O_2_ and demonstrates exceptional catalytic performance in the epoxidation of 1‐hexene. The newly formed hexa‐coordinated Ti(OSi)_2_(OH)_2_(H_2_O)TPA markedly enhances the activation of H_2_O_2_ significantly, and achieves a 1‐hexene conversion rate of 35.5%, surpassing that of untreated TS‐1 (18.0%). Furthermore, alkaline organic amine molecules can neutralize the acidicity of Si‐OH within the TS‐1, effectively inhibiting side reactions such as epoxide ring opening and resulting a high epoxide selectivity of 98.6%. When TPAOH and (NH_4_)_2_CO_3_ are employed to treat TS‐1 via the dissolution‐recrystallization process, abundant novel hexa‐coordinated Ti species Ti(OSi)_2_(OH)_4_ and internal voids can be formed.^[^ ^138^ ^]^ The high oxidation activity of Ti(OSi)_2_(OH)_4_ and internal voids increase the accessibility of Ti active species. The presence of Ti(OSi)_2_(OH)_4_ makes the catalytic performance of TS‐1 nearly twice that of conventional TS‐1, exhibits H_2_O_2_ conversion of 98.6% and glycol selectivity of 98.8% (Figure 20b).

Using alkaline reagents, such as TPAOH, EA to obtain TS‐1 containing highly coordinated titanium species via dissolution‐recrystallization post‐treatment is commonly employed method. Zhai et al.^[^ ^139^ ^]^ demonstrated economical synthesis of hollow TS‐1 free from anatase by utilizing recycled mother liquor as the post‐treatment agent through a dissolution‐recrystallization process. Through a co‐regulation study of kinetics and thermodynamics, it was found that a fascinating reversible transformation of titanium species occurred within TS‐1, revealing the microscopic mechanisms underlying the titanium dissolution‐reinsertion‐secondary dissolution processes during mother liquor recrystallization. The residual unsaturated silicon species in the mother liquor, together with dissolved Si species, synergistically induced the re‐incorporation of anatase TiO_2_, thereby preventing its formation.

However, up to now, the understanding of the mechanism of dissolution and recrystallization is still insufficient, the cost is high and the environmental problems are still serious. It is still a huge challenge to realize anatase‐free hollow TS‐1 with a controllable titanium microenvironment through dissolution and recrystallization for various catalytic reactions.

In the ammonoximation reaction system, the alkaline environment tends to erode the silica framework of TS‐1, resulting in desilication and the elimination of framework TiO_4_.^[^ ^139^, ^140^ ^]^ The surface of TS‐1 is covered with a substantial quantity of amorphous Ti and anatase TiO_2_. A dissolution‐recrystallization treatment is conducted on the deactivated TS‐1 using a solution of TPABr and ethanolamine.^[^ ^141^, ^142^ ^]^ During the dissolution phase, the Si and Ti species originally present in the deactivated TS‐1 are solubilized into the solution containing TPABr and ethanolamine, subsequently undergoing recrystallization. The extra‐framework Ti species are reintegrated into the framework, leading to a significant increase in the framework Ti content. Moreover, the high content of framework Ti species generated on the surface through recrystallization provides more opportunities for the reactant substrates to access the active centers.

During the dissolution‐recrystallization process of TS‐1, amorphous Si and Ti dissolve and subsequently recrystallization under the roles of various bases and templates. This process not only converts amorphous Ti into tetra‐coordinated framework Ti but also alters the coordination state of titanium and introduces newly active titanium species (defective Ti‐OH, TiO_5_, TiO_6_). These changes significantly boost their catalytic performance in oxidation reactions. Fundamentally, the dissolution‐recrystallization approach offers an effective means to circumvent the constraints of microporous mass transfer and enhance the accessibility of active Ti species. Throughout this process, a dynamic equilibrium is maintained between the dissolution and recrystallization of SiO_4_ and TiO_4_. This equilibrium enables the regulation of the synergistic interaction between the base and template, thereby facilitating the transformation of TiO_4_ into more active Ti species, such as penta‐coordinated Ti and hexa‐coordinated Ti titanium entities. Notably, alkali and template agents play pivotal roles in this transformation, however, the underlying mechanisms for the formation of more active titanium species remain poorly understood. It is necessary to delve into the kinetic and thermodynamic mechanisms during the dissolution‐recrystallization process and analyze the behavioral characteristics of active titanium species at different crystallization stages. By integrating advanced characterization techniques such as in situ X‐ray diffraction and scanning transmission electron microscopy, systematic experimental research is conducted to monitor in real‐time the changes and distribution states of titanium species during the dissolution‐recrystallization process. Meanwhile, DFT theoretical calculation are employed to simulate the interactions of active titanium species in the dissolution‐recrystallization environment, providing a theoretical basis for finding precise control methods.

### 3.4 Charge Regulation of Ti Active Center

In the TS‐1/H_2_O_2_ catalytic system, the electronic state of Ti species represents its inherent catalytic ability. The core mechanism behind the activation of H_2_O_2_ is attributed to the Lewis acidic nature of titanium, which enables it to coordinate in an unsaturated state and thereby accept lone pair electrons. Moreover, enhancing the positive charge of the Ti active centers is beneficial for improving its catalytic activity.

When Zn(Ac)_2_ is introduced the TPAOH‐treated TS‐1 process, silicon defect sites facilitate zinc incorporation into the framework, inhibiting TiO_4_ species' transformation into anatase TiO_2_.^[^ ^143^ ^]^ The altered charge distribution around Ti atoms in TS‐1‐Zn relaxes its structure, fostering the formation of a 5MR Ti‐OOH intermediate and enhancing its olefin epoxidation performance. Xue et al.^[^ ^144^ ^]^ prepared fluorinated TS‐1 (F‐TS‐1) by treating TS‐1 in a dimethyl sulfoxide solution of sodium fluoride under the assistance of microwave. F atom has strong electron‐withdrawing effect, and its introduction increases the positive charge of Ti active centers. F‐TS‐1 displays pronounced Lewis acidity and hydrophobicity, exhibiting superior catalytic performance in cyclohexanone ammoximation.

The electronic state of Ti species is a fundamental indicator of their intrinsic catalytic potential, and augmenting the positive charge on Ti active centers proves advantageous for enhancing their catalytic activity. Through micro – environmental modulation of Ti species, precise control over their catalytic performance can be attained by fine‐tuning their electronic characteristics.

Heteroatoms doping modification emerges as an ideal strategy for enhancing catalytic performance. The introduction of trivalent ions, such as Al^3+^, B^3+^, or ^3+^,^[^ ^145^ ^]^ not only boosts the number and strength of acid sites in TS‐1 but also improves the direct synthesis of ethylene glycol (EG) from ethylene and H_2_O_2_. This modification alters the local microenvironment surrounding Ti species, thereby modifying their electronic state and, consequently, their catalytic performance.

Simultaneously, the incorporation of metal heteroatoms enables efficient and stable catalysis through the synergistic effects of dual or multiple active sites.^[^ ^146^ ^]^

By adopting the approach of introducing heteroatoms to modify the local microenvironment of Ti species, this strategy can precisely regulate charge of Ti species and thereby achieve accurate control over their catalytic performance. Moreover, the incorporated heteroatoms can endow TS‐1 with dual or multiple sites synergistic effects, facilitating multi‐step or continuous reactions, and ultimately applying these advancements to the synthesis of more valuable fine chemicals. However, this method has a potential drawback that transition metal atoms may compete with Ti atoms for incorporation into the framework. This competition can lead to a decrease in content of framework Ti species. Consequently, further exploration is still required to utilize metal elements for tuning the electronic states of Ti and to prepare TS‐1 with dual/multiple active sites.

As shown in **Table** **1**, it summarizes the catalytic performance of TS‐1 containing different coordinated Ti active species in selective oxidation reaction. It has been observed that the coordination state of Ti within TS‐1 significantly influenced its catalytic performance in alkene epoxidation, with the order of hexa‐coordinated Ti > penta‐coordinated Ti > tetra‐coordinated Ti. Moreover, for Ti active species with the same coordination in TS‐1, a higher number of Ti‐OH is associated with enhanced catalytic activity. The same principle applies to both cyclohexanone ammoximation and the conversion of ethylene to ethylene glycol. Consequently, through the precise modulation of the generation of high‐coordinated Ti active species by employing diverse strategies, we can effectively foster the further advancement of selective catalytic oxidation reactions. In addition, the chemical environment of the titanium centers also influence the mechanisms in ammoximation, epoxidation, and hydroxylation. In the ammonoximation reaction system, the alkaline environment tends to erode the silica framework of TS‐1, resulting in the elimination of framework TiO_4_. Solvent plays a crucial role in affecting the reaction mechanism by involving hydrogen transfer process and facilitating the formation of hydrogen bond. Small molecules such as NH_3_, H_2_O, H_2_O_2_ can form high‐coordinated Ti active intermediates with Ti active species. These intermediates can reduce the energy barriers for H_2_O_2_ dissociation. Hence, improving the performance of catalytic reactions necessitates not only a high‐performance TS‐1 catalyst but also a more precisely controlled chemical reaction environment.

**Table 1:** Comparison of TS‐1 containing different Ti active species in selective oxidation.

Ti active species	Catalytic performances	TOF/TON	Catalytic reaction	Refs.
Ti(OSi)_3_(OH)_2_ and Ti(OSi)_4_	Utilization (H_2_O_2_) of 58.3% PO selectivity of 90.2%	753.4(mol H_2_O_2_/(h·mol Ti))	propene epoxidation	[63]
Ti(OSi)_4_	Utilization (H_2_O_2_) of 21.8% PO selectivity of 82.0%	156.8(mol H_2_O_2_/(h·mol Ti))	propene epoxidation	[63]
Ti(OSi)_2_(OH)_2_(H_2_O)_2_ and Ti(OSi)_4_	1‐hexene conversion rate of 33%	153	1‐hexene epoxidation	[100]
Ti(OSi)_4_	1‐hexene conversion rate of 22.5%		1‐hexene epoxidation	[100]
hydrogen‐bonded Ti species involving Ti(OSi)_3_(OH)(H_2_O) and Ti(OSi)_4_	1‐hexene conversion rate of 25%		1‐hexene epoxidation	[111]
TiO_4_ and TiO_6_	1‐hexene conversion rate of 26%		1‐hexene epoxidation	[111]
Ti(OSi)_3_OH and Ti(OSi)_4_	1‐hexene conversion rate of 44.9%	415	1‐hexene epoxidation	[132]
Ti(OSi)_4_	1‐hexene conversion of rate 19.9%	184	1‐hexene epoxidation	[132]
Ti(OSi)_2_(OH)_2_(H_2_O)TPA and Ti(OSi)_4_	1‐hexene conversion rate of 35.5%		1‐hexene epoxidation	[137]
Ti(OSi)_3_(OH)_2_, Ti(OSi)_3_(OH) and Ti(OSi)_4_	propylene conversion rate:≈23%	2.27(mol /(g·min)	propylene epoxidation	[20]
Ti(OSi)_2_(OH)_4_ and Ti(OSi)_4_	propylene conversion rate: ≈27%	5.44(mol /(g·min)	propylene epoxidation	[20]
Ti(OSi)_2_(OH)_2_(OH_2_)_2_ and Ti(OSi)_4_	propylene conversion rate: ≈16%	3.99(mol /(g·min)	propylene epoxidation	[20]
Ti(OSi)_4_	propylene conversion rate: ≈12%	1.49(mol /(g·min)	propylene epoxidation	[20]
Ti(OSi)_2_(OH)_4_ and Ti(OSi)_4_	cyclohexene conversion rate of 45.4%, epoxycyclohexane selectivity of 94.5%		epoxidation of cyclohexene	[135]
Ti(OSi)_4_	Cyclohexene conversion rate of 19.0%, epoxycyclohexane selectivity of 80.4%		epoxidation of cyclohexene	[135]
hydrogen‐bonded Ti species involving Ti(OSi)_3_(OH)(H_2_O) and Ti(OSi)_4_	allyl chloride conversion rate of 72%	340	allyl chloride epoxidation	[111]
TiO_4_ and TiO_6_	allyl chloride conversion rate of 32%	202	allyl chloride epoxidation	[111]
(SiO)_2_Ti (OH)_2_(H_2_O)_2_ and TiO_4_Ti(OSi)_4_	cyclohexanone conversion rate of 53% ammoximation selectivity of 98%		cyclohexanone ammoximation	[53]
Ti(OSi)_4_	cyclohexanone conversion rtae of 42% ammoximation selectivity of 19%		cyclohexanone ammoximation	[53]
Ti(OSi)_3_OH and Ti(OSi)_4_	the lifetime of 45 h		cyclohexanone ammoximation	[132]
Ti(OSi)_4_	the lifetime of 20 h		cyclohexanone ammoximation	[132]
Ti(OSi)_2_(OH)_4_ and Ti(OSi)_4_	Utilization (H_2_O_2_) of 98.6% and glycol selectivity of 98.8%		conversion of Ethylene to Glycol	[138]
TiO_4_	Utilization (H_2_O_2_) of 87.0% and glycol selectivity of 94.7%		conversion of Ethylene to Glycol	[138]

### 3.5 Deactivation of TS‐1

TS‐1 has been widely used in selective oxidation reactions, and during the reaction process, the catalyst inevitably undergoes deactivation. The deactivation typically occurs due to the inaccessibility of active species and a reduction in the number of active centers. The decline in Ti active centers can primarily be categorized into two types: irreversible deactivation and reversible deactivation. Regarding reversible deactivation, the presence of macromolecular polymers in the reaction system prevents the reaction substrate from smoothly accessing the Ti active centers within the molecular sieves, thereby reducing the number of Ti active centers participating in the catalytic reaction. For instance, following an industrial trial reaction lasting 1700 h, the TS‐1^[^ ^147^ ^]^ extrusion catalyst demonstrates signs of partial deactivation, primarily due to pore blockage and the occlusion of active sites by ethers or oligomers. Additionally, a minor loss of framework titanium is found to have a negligible impact on the catalyst's activity.

TS‐1 reversible deactivation, caused by the accumulation of heavy oligomers and low‐boiling adsorbates.^[^ ^148^ ^]^ The deactivation of TS‐1 with blocking agents can be regenerated through washing with H_2_O_2_ or high‐temperature calcination. Nevertheless, the regenerated TS‐1 exhibit reduced durability and initial selectivity for the irreversible hydrolysis of Ti─O─Si bonds when exposed to H_2_O_2_ solution, as well as increasing in surface OH groups and acidity.

An investigation into the causes of Hollow‐TS‐1 deactivation following the acetone ammoxidation process.^[^ ^149^ ^]^ XRD and UV–vis analyses of both fresh and deactivated Hollow‐TS‐1 samples reveal minimal changes, suggesting that the quantities of framework Ti and anatase TiO_2_ in the deactivated TS‐1 remain largely unchanged. Two primary mechanisms are proposed to explain the deactivation of the Hollow‐TS‐1 catalyst during the ketone ammoxidation reaction: 1) The adsorption of strongly basic by‐products amines onto the active sites result in a slow deactivation of the catalyst; 2) The accumulation of the by‐product 2,3‐dimethyl‐2,3‐dinitrobutane block the pore channels of the catalyst, leading to rapid deactivation.

It has been found no titanium atoms are leached from the TS‐1 framework during the H_2_O_2_‐mediated oxidation of styrene.^[^ ^150^ ^]^ Some Ti atoms in spent TS‐1 can transform from tetra‐coordinated to penta‐coordinated and hexa‐coordinated Ti species due to strong chemical adsorption of styrene, zaldehyde and benzaldehyde. Ti species in spent TS‐1 undergo changes in their coordination states.

Irreversible deactivation occurs in TS‐1 used in the commercial cyclohexanone ammoxidation process.^[^ ^151^ ^]^ The deactivated TS‐1 exhibit a reduced Si/Ti ratio dropping from 19.57 to 15.98. Additionally, it is observed that some of the framework Ti have transformed into amorphous TiO_2_. In the deactivated TS‐1 zeolite, both Brønsted and Lewis acid sites are detected, and these sites are attributed to the amorphous TiO_2_‐SiO_2_ nanoparticles situated on the external surface of the zeolite.

The deactivation of some TS‐1 catalysts is attributed to the change of microenvironment around the Ti active center. Such as the deactivation of TS‐1 caused by adsorption of organic molecules on the active center leads to the change of Ti coordination state. The deactivation of TS‐1 caused by the removal of tetra‐coordinated framework Ti and the transformation to TiO_2_. The deactivation of TS‐1 caused by the increase of acidity due to the increase of surface Si‐OH or Ti‐ groups. The deactivation reasons of the TS‐1/H_2_O_2_ system and the change of Ti active centers during the deactivation process are analyzed to provide a better perspective for improving the utilization of deactivated TS‐1 zeolite and for the development of structure‐stable catalysts. Furthermore, Integrating the TS‐1 oxidation system with the in situ electrocatalytic synthesis of hydrogen peroxide can enhance system stability and address deactivation challenges.^[^ ^152^ ^]^

## 4 Conclusion

Titanosilicate‐1 (TS‐1) has exhibited excellent catalytic performances for the production of many key chemical intermediates under mild reaction condition over the past 40 years. However, as their applications expand, the limitations of this catalytic material have become more apparent. Tetra‐coordinated framework Ti is widely regarded as the catalytic center for these oxidation reactions, while penta‐ and hexa‐coordinated Ti also exhibit enhanced catalytic activity in certain oxidation reactions. Tetra‐coordinated Ti species, Ti(OSi)_4_ or Ti(OSi)_3_(OH), can directly react with H_2_O_2_/H_2_O to generate various types of Ti peroxide species, including the Ti‐OOH 5‐MR, Ti‐(η^2^‐OO)‐3MR, and Ti(OSi)_3_OOH. High‐coordinated Ti species (penta‐ and hexa‐coordinated) exhibit a greater tendency to resemble similar Ti peroxide species in the presence of H_2_O_2_/H_2_O. All of Ti active species exert catalytic effect in various oxidation reactions by forming various types of active intermediates Ti peroxide species when reacting with H_2_O_2_. Besides the unique coordination characteristics of Ti, the microenvironment surrounding the active center including factors like the solvent and small molecules such as NH_3_ present in the reaction also influences catalytic mechanism and active intermediates. This occurs by affecting the binding between Ti active species and H_2_O_2_.

A series of top‐down or bottom‐up synthesis strategies aim to increase the amount of tetra‐coordinated framework titanium, inhibit anatase TiO_2_, and create a certain amount of highly coordinated titanium. 1) Methods for controlling and matching of the hydrolysis rates between silicon and titanium sources to achieve synthesis of TS‐1 with more framework Ti and anatase‐free. 2) Additionally, methods for modifying the microenvironment of active centers control crystallization mechanisms by adjusting pH, adding molecular modifiers, altering the crystallization route. These approaches effectively improve framework titanium, reduce anatase TiO_2_, and generate highly active coordinated titanium species. 3) Adding base and template during the dissolution‐reprecipitation process alters the microenvironment for TS‐1 reprecipitation. This affects not only the proportion of tetra‐coordinated Ti in TS‐1 but also plays a vital role in redistributing Ti species and generating new high‐coordinated Ti species. 4) The introduction of F and other atoms can change the electronic state of Ti active species in TS‐1, improve the positive charge of active center, and contribute to the improvement of catalytic activity. While numerous methods exist to effectively enhance the catalytic performance of TS‐1, it remains imperative to further streamline the synthesis process, optimize synthesis conditions, and explore novel synthesis approaches.

Exploration of TS‐1 catalysts with specific Ti species for specific reactions has broader industrial development potential. Clarify the catalytic mechanism of various active titanium species in different reactions, and accurately match highly active Ti species. Artificial intelligence (AI) and machine learning (ML) are steadily becoming the pivotal driving forces in catalytic research. By using the powerful data processing capability of AI to analyze spectroscopic data and kinetic information, it is feasible to accurately deduce the intermediates and transition states in catalytic reactions, thereby gaining profound insights into the reaction mechanisms of active centers. By using ML models to perform in‐depth analysis of the crystal structure and electronic properties of the catalysts, it is possible to accurately predict their key indicators, including activity and stability, thereby further exploring the intrinsic correlation between the synthesis parameters and the catalyst performance, and providing optimal conditions for the synthesis of highly efficient catalysts. Control the dimension of molecular sieves, synthesize 2D TS‐1 nanosheets, and achieve highly uniform and accessible titanium active species. By precisely adjusting the crystallization process and controlling the zeolite size, 2D TS‐1 nanosheets can be synthesized to achieve high uniformity and accessibility of the distribution of active Ti species. This uniformity is beneficial for fully utilizing their catalytic potential in oxidation reactions. Regulate the electronic state of Ti species, and accurately enhance catalytic performance. By adjusting the microenvironment of Ti species, its catalytic Heteroatom doping represents an effective strategy for local microenvironment modification, altering Ti electronic states and consequently optimizing catalytic activity. Design dual or multiple active sites, and explore multifunctionality of molecular sieves. Multi‐Site Catalyst Design Doping‐induced heteroatom incorporation endows TS‐1 with dual/multi‐site synergistic effects, enabling efficient and stable catalysis. Metal/metal compound nanoparticle‐coated TS‐1 zeolites provide abundant interface sites for tandem catalysis, while molecular sieve‐enzyme hybrids leverage complementary system advantages to achieve synergistic chemical‐enzymatic reactions. These multi‐active‐site architectures promote multi‐step or continuous reactions, advancing the synthesis of high‐value fine chemicals.

## Conflict of Interest

The authors declare no conflict of interest.
